# Lactation is Associated with Accelerated Postpartum Pelvic Floor Muscle Recovery in a Pregnant Simulated Birth Injury Model

**DOI:** 10.1002/advs.202600052

**Published:** 2026-06-09

**Authors:** Bianca L. Peña, Khushi M. Diwakar, Hillary K. Tran, Selena Cao, Celeste E. Lintz, Laila N. Hayes, Marianna Alperin, Karen L. Christman

**Affiliations:** ^1^ Shu Chien‐Gene Lay Department of Bioengineering University of California San Diego La Jolla California USA; ^2^ Sanford Consortium for Regenerative Medicine La Jolla California USA; ^3^ School of Biological Sciences University of California San Diego La Jolla California USA; ^4^ Department of Obstetrics, Gynecology, and Reproductive Sciences Division of Urogynecology and Reconstructive Pelvic Surgery University of California San Diego La Jolla California USA; ^5^ Sanford Stem Cell Institute La Jolla California USA

**Keywords:** birth injury, lactation, muscle regeneration, pelvic floor muscles, postpartum, pregnant

## Abstract

Healthy pelvic floor muscles (PFMs) are essential for proper pelvic floor function. The biggest risk factor for PFM dysfunction is injury sustained during vaginal childbirth, yet the factors that facilitate or impair PFM recovery from birth injury remain unknown. We aimed to assess the impact of the postpartum milieu in the presence and absence of lactation on PFM regeneration following simulated birth injury (SBI) in a pregnant rat model. We determined that lactating rats have a rapid increase in systemic immune markers in response to SBI, which contributes to rapid changes in PFM immune cell infiltration and decrease in pro‐inflammatory genes. PFMs in lactating rats have enrichment of anti‐inflammatory genes and larger newly formed myofibers that contribute to larger myofiber size after weaning. This suggests that lactation promotes an earlier anti‐inflammatory response in injured PFMs that allows for quicker myogenesis and myofiber hypertrophy.

## Graphical abstract

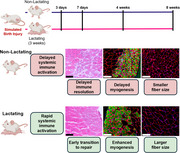

## 1 Introduction

Pelvic floor disorders, including urinary incontinence, fecal incontinence, and pelvic organ prolapse, are present in 30% of women worldwide, with some countries such as Italy, Switzerland, and Spain having rates as high as 50% in vaginally parous women, with many reporting symptoms that hinder their quality of life [1, 2]. One of the greatest risk factors for all pelvic floor disorders, especially pelvic organ prolapse, is pelvic floor muscle (PFM) dysfunction secondary to the maternal childbirth injury [3, 4, 5]. PFMs are integral to the supportive function of the female pelvic floor, as well as for maintaining continence and sexual function. The levator ani muscle complex of the human PFMs includes iliococcygeus and pubovisceralis, which is further divided into more lateral pubococcygeus and more medial puborectalis [6]. While imaging studies have been successfully deployed to assess PFM volumes and presence/absence of muscle avulsions in living women, it is difficult to directly measure changes in PFMs during childbirth. The computational 3D finite element models of human parturition demonstrate that the enthesis of the pubococcygeus muscle experiences the largest peak stress of ∼9.0 MPa and the highest stretch ratio of 4.64 compared to other PFMs, resulting in a high risk of mechanical injury [7]. To develop novel preventative and treatment strategies, the tissue, and cellular level response of PFMs to strains associated with vaginal delivery needs to be determined. Given the constraints associated with performing such studies in humans, especially in pregnant women, the use of preclinical models is essential.

Previous work from our and other research groups validated the rat model for the studies of the human PFMs [8, 9, 10, 11]. More specifically, it has been shown that the pubocaudalis muscle in the rat model is analogous to the pubococcygeus muscle in humans that undergoes the highest mechanical strains during vaginal childbirth [8, 9]. Previous studies from our group have demonstrated that during simulated birth injury (SBI), the rat pubocaudalis experiences strains similar in magnitude to those computed for the pubococcygeus [12]. Studies in the rat have demonstrated that pregnancy induces unique adaptations of PFMs by adding sarcomeres in series to increase fiber length and prepare for the delivery of pups [13]. Further work studying how different vaginal distention volumes that mimic parturition‐associated strains impact PFMs in non‐pregnant and pregnant rats concluded that pregnancy‐induced adaptations attenuate sarcomere hyperelongation and protect against mechanical muscle injury, with large distention volumes required to surpass these adaptations in pregnant rats [12]. Our previous studies in a non‐pregnant rat model conclude that SBI causes impairment in muscle anabolism, a sustained inflammatory response, and long‐term atrophy and fibrosis in the most translationally relevant pubocaudalis portion of the PFM complex [14]. While these findings are informative, the non‐pregnant model does not recapitulate the hormonal milieu associated with pregnancy, parturition, and lactation.

Published clinical studies have not identified a correlation between breastfeeding and pelvic floor disorder development, but these focused on the development of symptomatic pelvic floor disorders decades after childbearing and lacked tissue‐level examination of PFMs. Thus, neither the extent of muscle injury, other than the extreme avulsion phenotype, nor the degree of (non)recovery can be ascertained in these epidemiological investigations [15, 16]. Importantly, postpartum women have a sharp decrease in estrogen following parturition, with physiological hypoestrogenemia continuing throughout lactation [17]. Systemic estrogen remaining low during lactation could have implications for PFM recovery as estrogen is known to aid in the regeneration of skeletal muscles [18, 19, 20]. Several studies have shown that high levels of estrogen can accelerate and control muscle growth, regeneration, and muscle stem cell behavior [20, 21, 22, 23, 24]. However, these studies focus on appendicular muscles in ovariectomy/post‐menopausal models or estrogen receptor knock‐out models and do not study the postpartum milieu and PFMs. Despite this well‐known state of hypoestrogenemia and the impact of vaginal delivery on PFMs, the effect of lactation on PFM healing postpartum has not been explored. Our most recent work examined how lactation affects muscle stem cells postpartum in the presence and absence of birth injury. Without a birth injury, muscle stem cell proliferation is blocked by lactation [25]. However, birth injury negated the inhibitory effect of lactation on muscle stem cells, illustrating that birth injury overcomes the systemic inhibitory effect of lactation when compared to uninjured postpartum rats [25]. While this study focused on muscle stem cells, the impact of lactation on the systemic immune response and long‐term PFM healing after birth injury remains unknown. In the current study, we aimed to use a translationally relevant pregnant rat model to determine the impact of SBI, parturition, and lactation on acute and long‐term PFM regeneration. We initially hypothesized that lactation may hinder PFM regeneration following birth injury due to the hypoestrogenic state.

## 2 Results

### 2.1 Systemic Hormones Vary with Lactation and Weaning in Postpartum Rats Following Birth Injury

**FIGURE 1:**
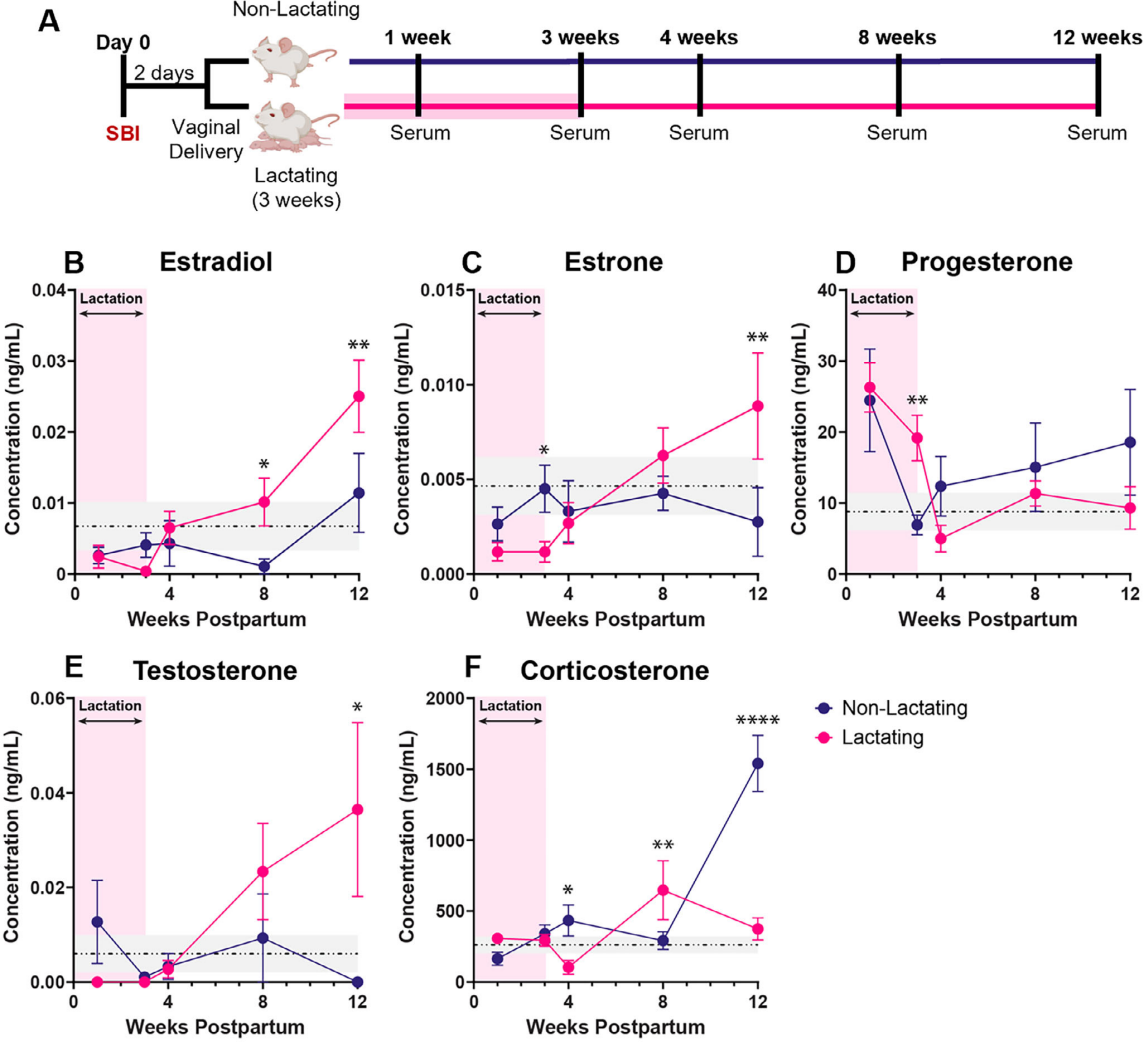
Systemic hormones change with lactation and weaning in postpartum rats. (A) Experimental summary for serum extraction postpartum for panels B–F. Summary serum measurements postpartum of (B) Estradiol, (C) Estrone, (D) Progesterone, (E) Testosterone, and (F) Corticosterone. All graphs represented as mean ± SEM. Gray shaded region represents the mean ±SEM non‐pregnant uninjured control values for each hormone. Non‐lactating measurements are shown in purple and lactating measurements are shown in pink, with pink highlighted regions indicating active lactation periods. All graphs include hormone measurements from 1wk (*n* = 9), 1 wk + Lac (*n* = 16), 3wk (*n* = 17), 3wk + Lac (*n* = 18), 4wk (*n* = 5), 4wk + Lac (*n* = 12), 8wk (*n* = 7), 8wk + Lac (*n* = 11), 12wk (*n* = 6), and 12wk + Lac (*n* = 6) rats. Two‐way ANOVA was used followed by post hoc pairwise comparisons using Sidak multiple comparison tests, *P<0.05, **P<0.01, ****P<0.0001 when comparing lactating to non‐lactating rats at each time point. SBI: Simulated birth injury.

We aimed to characterize the differences between circulating hormones in postpartum rats and how these hormone changes contribute to local pubocaudalis injury response following SBI. Serum was collected from non‐pregnant uninjured controls and at 1‐, 3‐, 4‐, 8‐, and 12‐weeks postpartum to measure hormones in non‐lactating and lactating rats who were subjected to SBI prior to delivery (Figure 1A). Non‐lactating rats had pups removed immediately postpartum while lactating rats continued lactation for 3 weeks until weaning of pups occurred following the natural weaning timeline (Figure 1A).

Estradiol, a primary estrogen isoform in reproductive age females, was persistently low throughout the 3‐week lactation period compared to non‐lactating rats, consistent with the physiological hypoestrogenemia in breastfeeding women (Figure 1B). Estradiol levels rapidly increased in the lactating group after weaning at 3‐weeks postpartum and continued to rise up to 12‐weeks. Estrone, a weak estrogen typically prevalent in postmenopausal women, presented similar trends as estradiol (Figure 1C). Progesterone, a hormone that is known to regulate muscle mass [26], was significantly higher in lactating rats at 3‐weeks postpartum (P = 0.002, Figure 1D). Testosterone, known to cause muscle hypertrophy and stimulate muscle protein synthesis [27, 28] was low in lactating rats up to 4‐weeks postpartum (Figure 1E). Corticosterone, the primary glucocorticoid stress hormone in rats equivalent to human cortisol [29, 30], differed across groups primarily after weaning at 3‐weeks (Figure 1F). To determine if there were acute stress differences between groups due to pup removal, corticosterone was also measured 3‐days after SBI, 24 hours after pup removal, showing no significant difference between groups (Figure S1). Corticosterone decreased rapidly in lactating rats after weaning pups at 3‐weeks postpartum and fluctuated around non‐pregnant levels thereafter. These data illustrate that lactation induces a unique hormonal milieu throughout and after the 3‐week lactation period.

### 2.2 Lactation Induces a Unique Systemic Immune Response Immediately Postpartum

**FIGURE 2:**
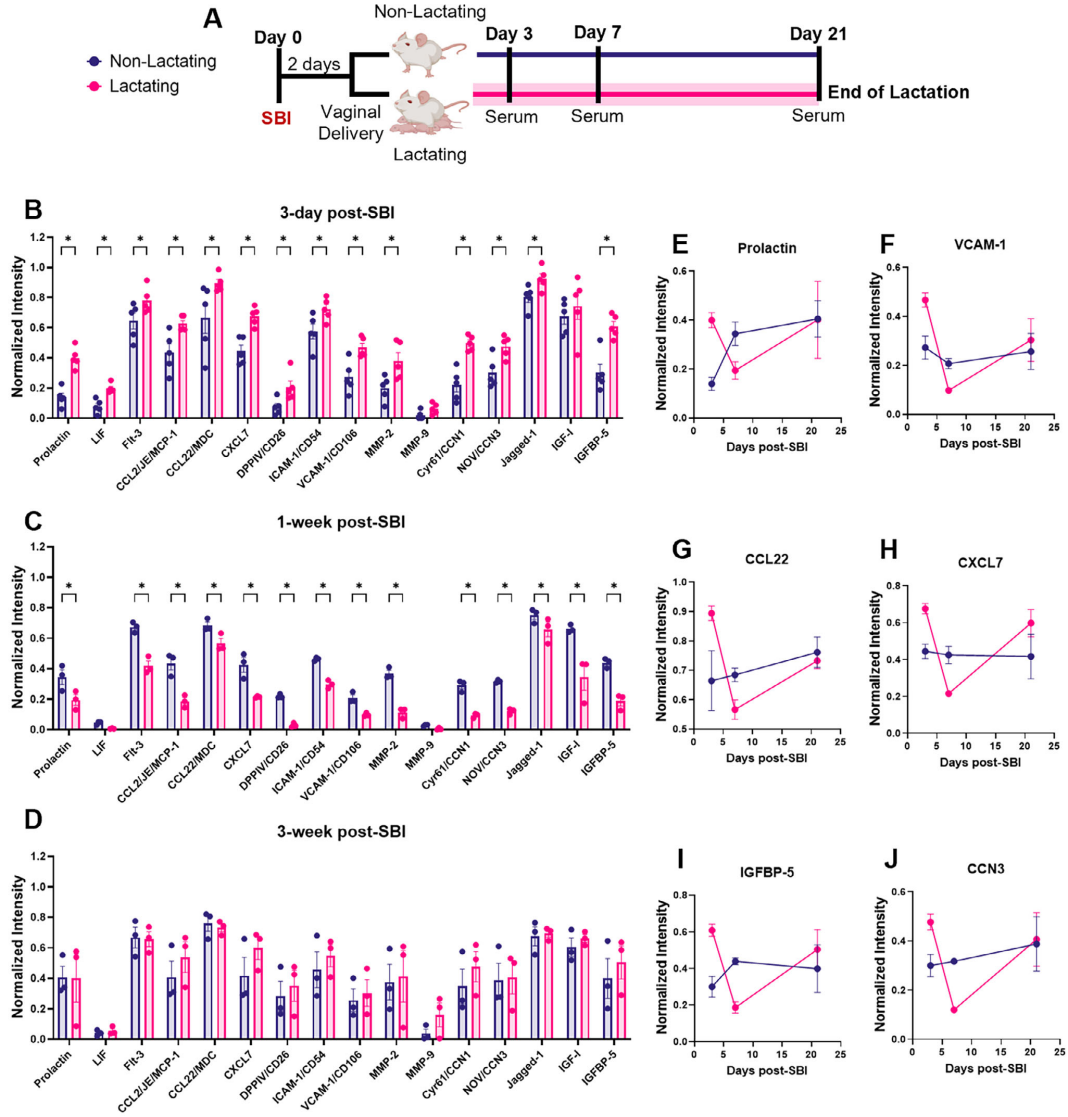
Systemic cytokines measured after birth injury show lactation induces a unique immune response immediately postpartum. (A) Experimental summary for serum extraction postpartum for panels B–J. (B) Bar graph representing systemic immune changes in serum extracted at 3‐days post‐SBI, 1‐day postpartum, (C) 1‐week post‐SBI and (D) 3 weeks post‐SBI. (E–J) Serum summary measurements depicted across time for (E) Prolactin, (F) VCAM‐1, (G) CCL22, (H) CXCL7, (I) IGFBP‐5, and (J) CCN3. 3d SBI (*n* = 5), 3d SBI + Lac (*n* = 5). 1wk SBI, 1wk SBI + Lac, 3wk SBI, and 3wk SBI + Lac each had *n* = 6 biological replicates pooled into *n* = 3 technical replicates. All graphs are shown as mean ± SEM. Non‐lactating measurements are shown in purple and lactating measurements are shown in pink. Two‐way ANOVA was performed, followed by a two‐stage linear step‐up procedure of Benjamini, Krieger, and Yekutieli with a false‐discovery rate of 0.05, *P<0.05. SBI: Simulated birth injury.

We next sought to determine how the systemic immune response changes with or without lactation via serum extracted at 3, 7, and 21 days after SBI, which was performed in late‐pregnant rats (Figure 2A). All animals for the 3‐day time point were monitored and sacrificed 24 h postpartum, with a complete 24‐h period lactating or not‐lactating (Figure 2A). The 79 different analytes from a Rat XL Proteome Profiler kit were categorized by function (Figures S2–S4). A shortlist of the 16 top cytokines that had large shifts related to lactation and immune regulation is displayed (Figure 2B–D). To better characterize how these cytokines change throughout the 3‐week lactation period, we also examined temporal patterns of cytokines unique to lactation that contribute to myogenesis and immune cell recruitment (Figure 2E–J).

At 3‐days after SBI (1‐day postpartum), the serum of lactating rats had an enrichment of many immune cell recruitment and adhesion markers (CCL2, CCL22, CXCL7, VCAM‐1, ICAM‐1, and CCN1), immune cell activation and migration markers (Flt‐3 ligand, Jagged‐1, CCN3, and DPPIV), and the anti‐inflammatory cytokine, prolactin – the hormone responsible for milk production (Figure 2B). While IGF‐1 levels did not differ across groups, IGFBP‐5 was higher in lactating rats. IGFBP‐5 influences cell proliferation and differentiation, binds to laminin and collagen to promote cell adhesion and migration, and stimulates myogenesis [31]. Despite only 24 h of lactation, immediate differences in immune cell activation and migration markers that can influence immune cell recruitment to the site of injury were observed.

Many of the proteins that were higher in lactating compared to non‐lactating rats at 3 days decreased by 7‐days after SBI (Figure 2C). The immune cell recruitment and adhesion markers, immune cell activation and migration markers, prolactin, and IGFBP‐5 were higher in non‐lactating rats compared to lactating rats at 7‐days. This indicates that the 7‐day non‐lactating rats underwent a similar systemic immune response to that which the lactating rats experienced at 3‐days.

At 21‐days after injury, there were no longer any differences across all 79 proteins detected with or without lactation (Figure 2D and Figure S4). This demonstrated that while there were different acute immune responses with lactation, these differences diminished by the end of the lactation when pups were weaned at 3‐weeks. Importantly, when describing the temporal cytokine changes, the lactating group had higher immune cell recruitment, adhesion, and activation marker values at 3‐days post‐SBI that decreased drastically by 7‐days (Figure 2E–J). Lactating rats experienced profound changes in cytokines throughout the lactation period whereas the non‐lactating rats had consistent levels of cytokines (Figure 2E–J). Overall, lactating rats had an accelerated systemic immune response compared to non‐lactating rats.

### 2.3 Lactating Rats Show Rapid Immune Cell Infiltration and Early Signs of Myogenesis at Acute Time Points

Given the systemic immune differences seen at 3, 7, and 21 days after SBI, we aimed to assess how these circulating factors impact the regenerative response of the injured pubocaudalis muscle. Pubocaudalis muscles were cryosectioned for analysis of CD45^+^ immune cells, fiber cross‐sectional area, and embryonic myosin heavy chain‐positive (eMyHC^+^) fibers. eMyHC is commonly used as a marker of newly regenerated myofibers with cross‐sectional area of eMyHC^+^ fibers being an indicator of size progression to a mature myofiber [32, 33].

**FIGURE 3:**
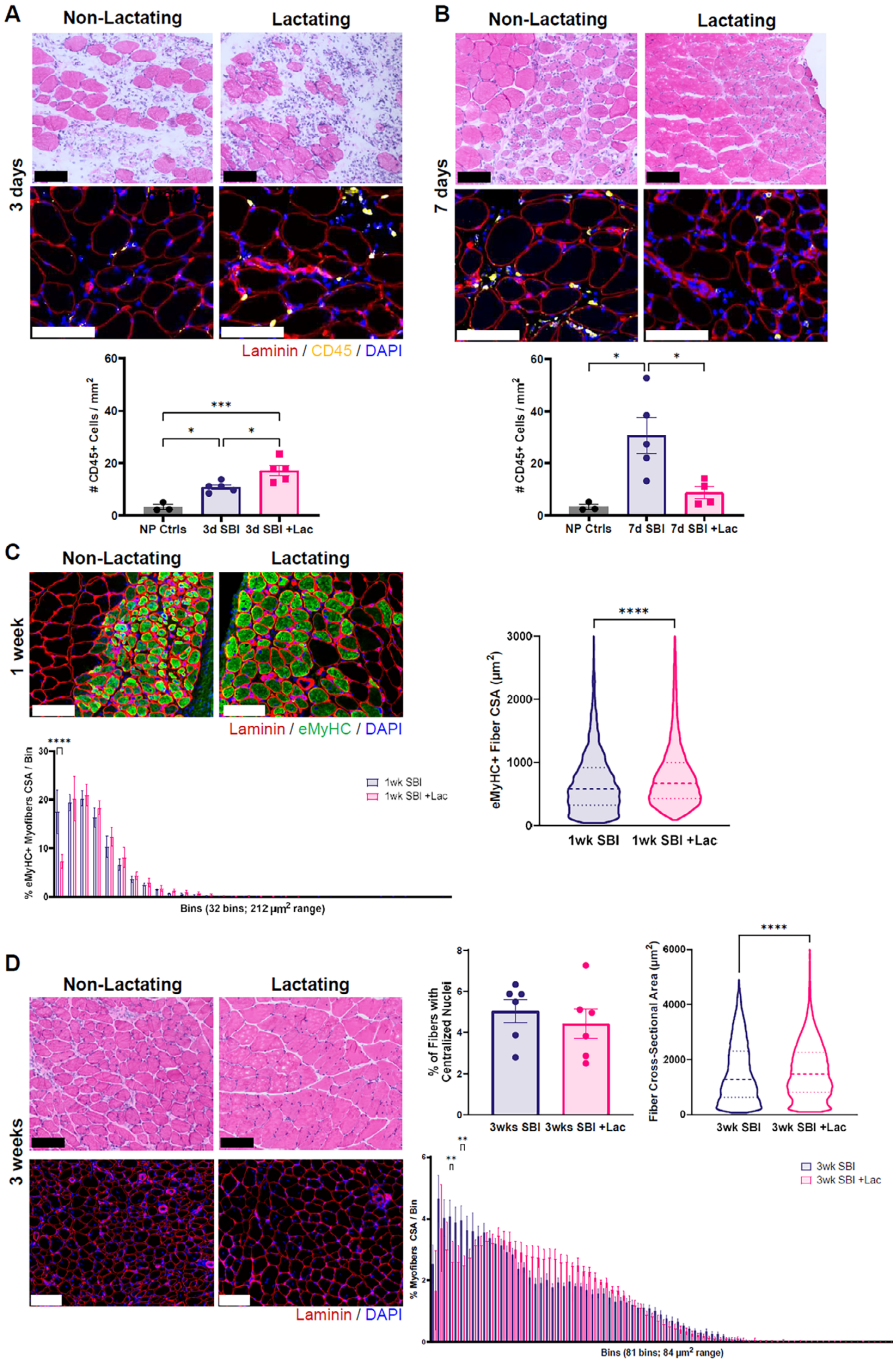
The pubocaudalis muscles of lactating rats have rapid immune cell infiltration and early signs of myogenesis at acute time points. H&E and immunofluorescent staining against laminin, CD45, and DAPI in (A) 3‐day and (B) 7‐day post‐SBI pubocaudalis muscles. Bar graphs representing quantification of CD45^+^ cells in non‐pregnant controls (NP Ctrls, *n* = 3) and non‐lactating and lactating samples at (A) 3‐day SBI (*n* = 5) and (B) 7‐day SBI samples (*n* = 5). (C) Immunofluorescent staining against laminin, eMyHC, and DAPI in 1‐week post‐SBI non‐lactating and lactating samples. Graphical representations of eMyHC+ myofiber cross‐sectional area distribution in 1wk SBI and 1wk SBI + Lac animals. 1wk SBI (*n* = 6), 1wk SBI + Lac (*n* = 6). (D) H&E and immunofluorescent staining against laminin and DAPI in 3 weeks post‐SBI non‐lactating and lactating samples. Bar graph representing quantification of percentage of fibers with centralized nuclei from laminin and DAPI images in 3wk SBI and 3wk SBI + Lac animals. Graphical representations of myofiber cross‐sectional area distribution in 3wk SBI and 3wk SBI + Lac animals. 3wk SBI (*n* = 6), 3wk SBI + Lac (*n* = 6). All bar graphs are shown as mean ± SEM. Scale bars are 100µm. For CD45 analysis, one‐way ANOVA with Tukey's multiple comparisons was performed. For binning of myofibers, two‐way ANOVA followed by post hoc pairwise comparisons using Sidak multiple comparison test was used. For overall myofibers comparisons, the Mann‐Whitney test was used. *P<0.05, **P<0.01, ***P<0.001, ****P<0.0001. NP: Non‐pregnant uninjured; SBI: Simulated birth injury; SBI +Lac: Simulated birth injury + lactation; eMyHC: embryonic myosin heavy chain; DAPI: 4′,6‐diamidino‐2‐phenylindole; CSA: cross‐sectional area.

We performed immunohistochemistry for CD45 to characterize immune cell infiltration to the injured pubocaudalis muscles (Figure 3A,B). Immune cell infiltration is used in many wound healing models to measure inflammation and determine temporal changes of the immune response [34]. We determined that lactating rats have a higher density of CD45^+^ cells 3‐days after injury, suggesting that lactation contributed to rapid immune cell migration to the local region of injury (Figure 3A). At 7‐days post‐SBI, the number of CD45^+^ cells decreased in lactating rats to non‐pregnant uninjured control values (Figure 3B). In contrast, non‐lactating rats experienced an increase in CD45^+^ cells at 7‐days, with values remaining significantly higher than non‐pregnant uninjured healthy controls. Given these changes in CD45^+^ cells, we also evaluated CD68^+^ macrophages and CD163^+^ M2‐like macrophages via immunohistochemistry (Figure S5) but found no significant differences between non‐lactating and lactating animals. Thus, lactating rats had an accelerated infiltration of CD45^+^ immune cells 3‐days after SBI, with a prompt decrease by 7‐days.

Cross‐sectional area analysis of eMyHC^+^ myofibers indicated that the pubocaudalis muscle of lactating rats had larger eMyHC^+^ fibers at 1‐week post‐SBI (Figure 3C). Non‐lactating rats had a significantly larger percentage of small fibers, specifically in the smallest bin range of 38–250 µm^2^ (Figure 3C). eMyHC+ myofibers are driven by differentiated muscle stem cells that can be measured via myogenin (MyoG^+^). Immunofluorescence of MyoG^+^ cells at 3‐days after SBI illustrated that lactating animals have significantly more MyoG^+^ cells than non‐lactating animals at this time point (Figure S6). These results demonstrated that lactating rats had an early increase of myoblasts that could be driving the larger, newly formed myofibers at 1‐week when compared to non‐lactating rats.

Fiber cross‐sectional area at 3‐weeks after SBI was larger in lactating compared to non‐lactating rats (Figure 3D). This demonstrated that 3 weeks after injury, non‐lactating rats still had small myofibers that had not fully matured. However, when assessing the percentage of fibers with centralized nuclei as a measurement of active regeneration, non‐lactating and lactating rats were not significantly different (Figure 3D). Immunohistochemistry analysis at 3‐days, 1‐week, and 3‐weeks after SBI corroborate the systemic immune response and myogenesis markers detected systemically.

### 2.4 PFMs in Lactating Rats are Enriched With Anti‐Inflammatory Genes and Regulatory T Cells Acutely AfterSBI

**FIGURE 4:**
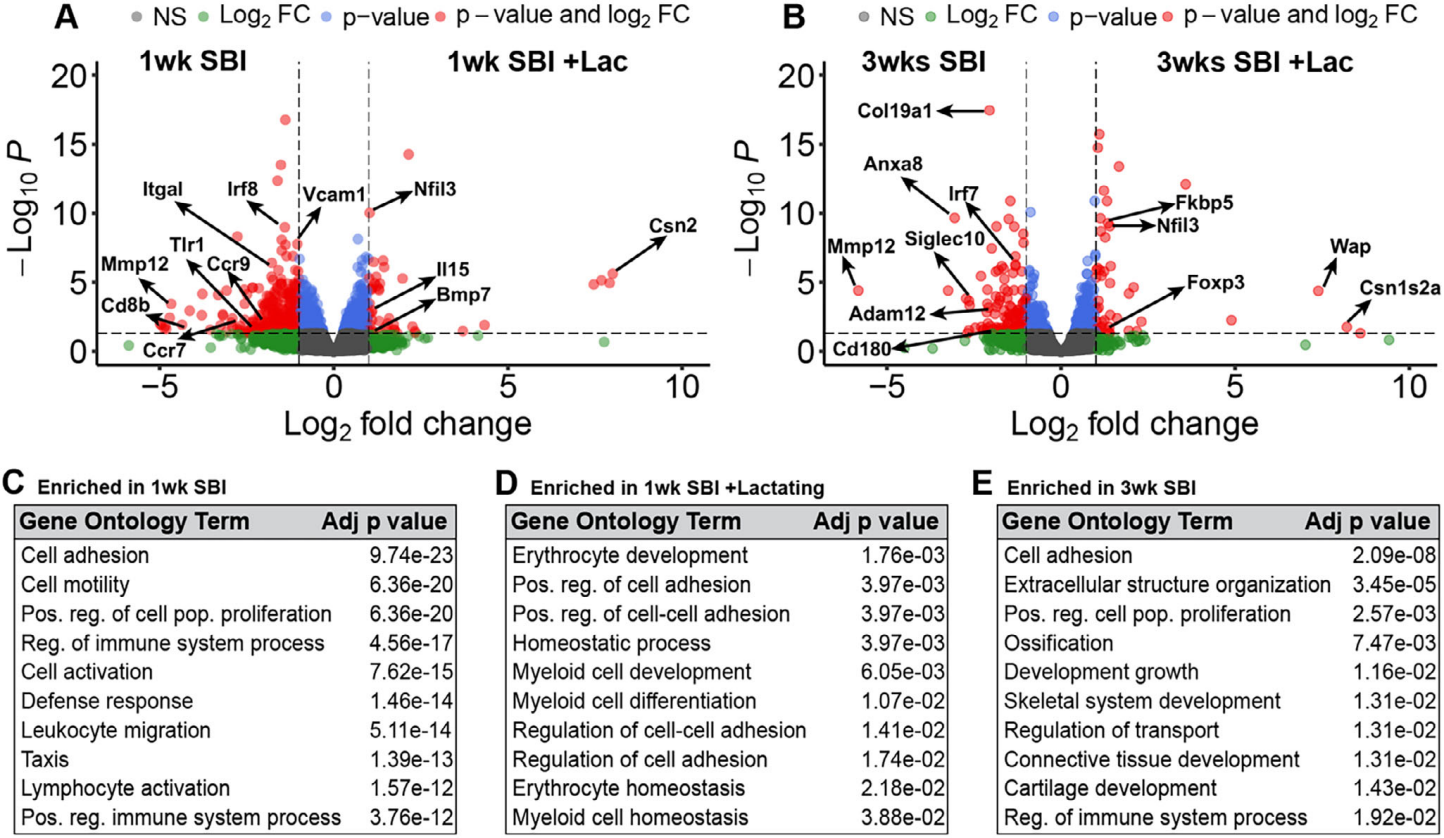
Lactation stimulates anti‐inflammatory genes and regulatory T cells while reducing pro‐inflammatory genes acutely after SBI. (A) Bulk‐RNA sequencing was performed on 1‐week SBI (left) and 1‐week SBI + Lac (right) pubocaudalis muscle and is represented in a volcano plot. (B) Bulk‐RNA sequencing was performed on 3‐week SBI (left) and 3‐week SBI + Lac (right) pubocaudalis muscle and is represented in a volcano plot. Red dots represent differentially expressed genes. (C) Top GO terms for upregulated genes in 1wk SBI animals are displayed compared to down‐regulated genes in 1wk SBI + Lac animals. (D) Top GO terms for upregulated genes in 1wk SBI + Lac animals are displayed compared to down‐regulated genes in 1wk SBI animals. (E) Top GO terms for upregulated genes in 3wk SBI animals are displayed compared to down‐regulated genes in 3wk SBI + Lac animals. All groups had biological replicates of *n* = 6 pooled into *n* = 3 technical replicates during sample preparation.

To further understand the impact of SBI on PFMs throughout lactation, we examined changes in gene expression with an unbiased bulk‐RNA sequencing approach. The pubocaudalis muscle was harvested at 1‐ and 3‐weeks after SBI for RNA isolation. Genes were analyzed with an established Bioconductor DESeq2 pipeline with P<0.05 and log2FoldChange>1 cutoffs to determine differentially expressed genes (Tables S1–S4) and are displayed on volcano plots (Figure 4A,B). Differentially expressed genes were further evaluated with Gene Ontology Enrichment Analysis for pathway characterization (Figure 4C–E).

At 1‐week after SBI, pubocaudalis muscles of non‐lactating rats were enriched with genes regulating the immune response (Figure 4A,C). Non‐lactating rats had upregulation of genes associated with adhesion and migration of immune cells (e.g. *Itgal, Itgax, Sele, Cx3cr1, and Vcam1*), T cell trafficking and activation (e.g. *Ccr9, Cd8b, Cxcr3, and Cd28*), myeloid cell markers (e.g. *Clec9a, Clec4a, Irf8, and Cd33*), and pro‐inflammatory markers (e.g. *Ccr7, C1qtnf3, Nfkbiz, and Clec7a*). Overall, the pubocaudalis muscle in non‐lactating rats at 1‐week after SBI had more of a pro‐inflammatory response that remained unresolved, as seen by defense response and overall immune cell activation pathways still very prominent in comparison to lactating rats (Figure 4C). In contrast, lactating rats had notable upregulation of anti‐inflammatory and regulatory T cell markers (e.g. *Bmp7, Alox15, Nfil3, and Il15*) (Figure 4A,D). Lactating rats demonstrated an early decrease of pro‐inflammatory markers with myeloid cell development, differentiation, and homeostatic pathways (Figure 4D).

At 3‐weeks after SBI, pro‐inflammatory genes (e.g. *Slamf9, Irf7, and Adam12*), immune cell activation markers (e.g. *Cd28, Cd180, and Coro1a*), and myofibroblast response markers (e.g. *Col19a1, Col6a5, Fap, Col5a3, and Thbs3*) continued to be upregulated in non‐lactating compared to lactating rats (Figure 4B,E). Non‐lactating rats had pathway enrichment of skeletal system development, connective tissue development, and regulation of immune system response, tied to their upregulation of various collagens and immune cell markers(Figure 4E). While 3‐week lactating rats had no enriched pathways based on differentially expressed genes, there was still an upregulation of Treg genes *Foxp3* and *Nfil3* (Figure 4B). Lactating rats at 3‐weeks also had upregulation of *Fkbp5*, which interacts with the mTOR pathway to influence protein synthesis, cell growth, and stress response. The pubocaudalis of lactating rats also had upregulation of *Wap* and *Csn1s2a*, whey acidic protein and casein‐alpha, both encoding proteins secreted in milk. WAP‐domain proteins are known to limit maladaptive tissue damage and promote tissue repair [35]. Taken together, transcriptional analysis demonstrated that lactating rats had downregulation of pro‐inflammatory genes, upregulation of anti‐inflammatory genes, and upregulation of Tregs, which control inflammation and maintain homeostasis.

### 2.5 Lactating Rats Present Larger Myofibers and Ongoing Regeneration After Weaning

**FIGURE 5:**
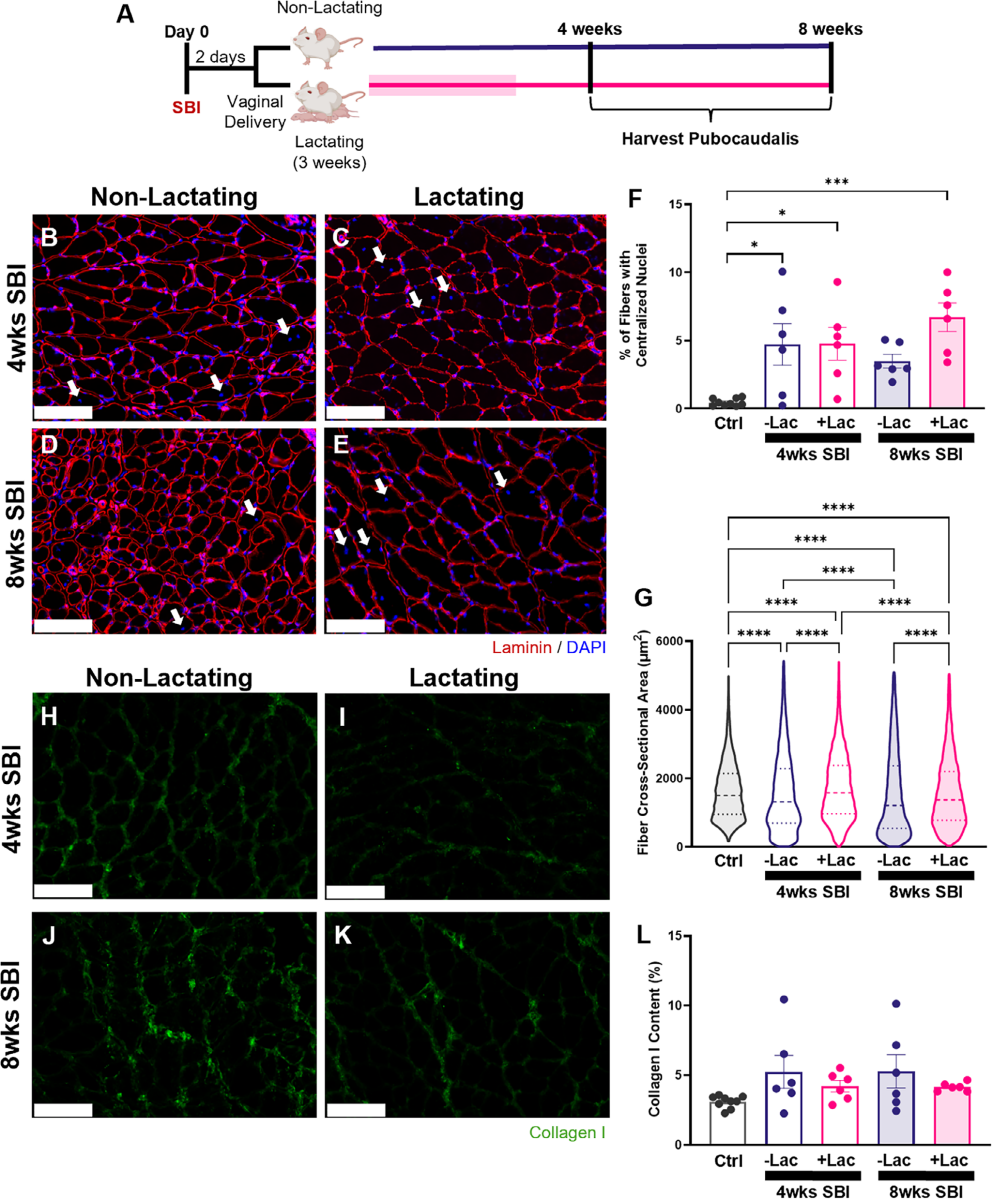
Lactation contributes to larger myofiber size and ongoing regeneration after weaning of pups. (A) Experimental summary of panels B–L. (B–D) Immunofluorescent staining against laminin and DAPI in (B) 4‐week post‐SBI, (C) 4‐week post‐SBI + Lac, (D) 8‐week post‐SBI, and (E) 8‐week post‐SBI + Lac pubocaudalis. (F) Bar graph representing quantification of percentage of fibers with centralized nuclei from laminin and DAPI images in healthy uninjured control, 4wk SBI, 4wk SBI + Lac, 8wk SBI, and 8wk SBI + Lac animals. (G) Graphical representation of myofiber cross‐sectional area distribution in healthy uninjured control, 4wk SBI, 4wk SBI + Lac, 8wk SBI, and 8wk SBI + Lac animals. (H–K) Immunofluorescent staining against collagen I in (H) 4‐week post‐SBI, (I) 4‐week post‐SBI + Lac, (J) 8‐week post‐SBI, and (K) 8‐week post‐SBI + Lac pubocaudalis. (L) Bar graph representing percentage of positive collagen I content in healthy uninjured control, 4wk SBI, 4wk SBI + Lac, 8wk SBI, and 8wk SBI + Lac animals. Scale bars are 100µm. All bar graphs are shown as mean ± SEM. Healthy uninjured controls (*n* = 9), 4wk SBI (*n* = 6), 4wk SBI + Lac (*n* = 6), 8wk SBI (*n* = 6), 8wk SBI + Lac (*n* = 6). For analysis of centralized nuclei, one‐way ANOVA was performed with the Sidak multiple comparison test. For analysis of fiber CSA, Kruskal‐Wallis test was performed followed by Dunn's multiple comparisons test. For analysis of collagen I content, one‐way ANOVA was used with Tukey's multiple comparisons test. *p<0.05, ***p<0.001, ****p<0.0001. SBI: Simulated birth injury; Ctrl: Healthy uninjured control; ‐Lac: Non‐lactating; + Lac: Lactating.

To understand the longer‐term impact of the 3‐week long lactation period, we harvested pubocaudalis at 4‐ and 8‐weeks after SBI, i.e. 1 and 5 weeks after cessation of lactation (Figure 5A). We assessed the impact of lactation and SBI with quantification of fibers with centralized nuclei, fiber cross‐sectional area, and collagen content. Non‐pregnant and late‐pregnant controls did not differ in the above readouts and were thus combined into one group to demonstrate if PFMs returned to a healthy uninjured phenotype.

When comparing 4 weeks post‐SBI rats to healthy uninjured controls (Ctrl), both non‐lactating and lactating groups had signficantly higher percentages of fibers with centralized nuclei (both P = 0.01, Figure 5F). The high number of fibers with centralized nuclei indicates ongoing regeneration and follows a pattern previously seen in non‐pregnant SBI models [14]. By 8‐weeks, the proportion of regenerating myofibers remained significantly higher in the lactating group compared to uninjured controls (P = 0.0002, Figure 5F). At 4‐weeks after SBI, the pubocaudalis of non‐lactating rats was enriched with smaller fibers while lactating rats had larger fibers compared to uninjured controls (P<0.0001 for both, Figure 5G). Non‐lactating rats remained enriched with smaller fibers with a lower median and mean rank at 8‐weeks when compared to uninjured controls and non‐lactating rats at 4‐weeks (Figure 5G). Pubocaudalis was still enriched with larger fibers 8‐weeks after injury in lactating rats compared to non‐lactating rats that have persistent smaller fibers. While the median fiber area decreased in 8‐weeks lactating rats when compared to 4‐weeks lactating rats, this may be due to the ongoing regeneration seen by high percentage of centralized nuclei. To determine if SBI in late‐pregnant rats induced changes in intramuscular ECM content we quantified collagen I, the main component of intramuscular fibrosis. Collagen I content did not differ across groups due to injury or due to lactation (Figure 5H–L). Although SBI in late‐pregnant rats did not induce long‐term fibrosis, at 4‐ and 8‐ weeks lactating rats sustained larger fibers and had prolonged regeneration compared to non‐lactating rats.

## 3 Discussion

In this study we investigated the systemic and local impact of lactation on pelvic floor muscle regeneration. Previous work focused on assessing the PFM injury response in non‐pregnant rats to characterize long‐term outcomes but lacked the translationally relevant component of postpartum lactation. Given that vaginal delivery is the leading risk factor for PFM injury [3], factors that could impact postpartum PFM recovery need to be investigated to develop strategies to potentiate constructive recovery and decrease the risk of postpartum PFM dysfunction and associated pelvic floor disorders. Lactation significantly impacts the postpartum systemic milieu, redirecting maternal metabolic needs to milk production [36]. Lactation also sustains a postpartum hypoestrogenic state that can impact the regeneration of skeletal muscles [24, 36, 37].

As the first step, we confirmed that systemic steroid hormones vary between non‐lactating and lactating rats, particularly estradiol, progesterone, and testosterone. We also found a rapid (within 24 h postpartum) systemic increase of cytokines involved in immune cell recruitment, adhesion, and activation in lactating rats, whereas non‐lactating rats do not demonstrate the analogous rise in these parameters. In parallel, at the tissue level, the acute expression of pro‐inflammatory genes is downregulated in PFMs following SBI in lactating compared to non‐lactating animals, consistent with the decrease in immune infiltrate identified in tissue slices. These changes likely contribute to the accelerated regeneration of injured PFMs in lactating rats, resulting in larger myofibers long term.

Systemically, estrogens and testosterone are low during lactation. Estradiol has been studied thoroughly in the skeletal muscle field and contributes to healthy muscle strength and regeneration, maintenance of muscle stem cell populations, and myogenesis [19, 20, 22, 24]. However, these studies focus on chemically induced injury to limb muscles in ovariectomized or estrogen receptor knock‐out animal models to depict estrogen loss in menopause, which is not analogous to our postpartum lactation model [19, 20, 22, 38]. While the lactating animals have lower estradiol and estrone values compared to the non‐lactating animals during the 3‐week lactation period, other hormones impacted by lactation, such as prolactin and progesterone, remain high. Prolactin – a polypeptide hormone synthesized and secreted by the anterior pituitary gland, is substantially impacted by lactation [39]. This hormone is required for the growth and development of the mammary glands, synthesis of milk, and maintenance of milk secretion [39]. Prolactin is also known to play a role in humoral and cellular immune responses and can influence the response to injury [40]. Studies have shown that prolactin regulates immune homeostasis via toll‐like receptors by activating cytokine signaling and downregulating inflammatory mediators [40]. Thus, high prolactin levels at the start of lactation could be mediating the rapid increase in systemic anti‐inflammatory cytokines and chemokines and contributing to immune cell recruitment, adhesion, and activation observed in the lactating animals 24 h postpartum. With high serum levels of VCAM‐1, ICAM‐1, CCL2, CCL22, CXCL7, and Flt‐3 ligand, lactating rats, compared to non‐lactating rats, express more leukocyte recruitment, adhesion, and activation, with an emphasis on neutrophils, regulatory T cells, and dendritic cells [41, 42]. CCL22 could accelerate muscle recovery in lactating rats by aiding in Treg recruitment, facilitating cell‐to‐cell contact with dendritic cells, and increasing the number of neutrophils [41]. Further, CCL2 is needed for adequate acute skeletal muscle repair post injury; the high CCL2 in lactating rats is thus potentially accelerating the regeneration timeline [43]. This derivation is supported by the findings in limb muscles, with CCL2 aiding in recruiting monocytes and macrophages to injured muscle and global deletion of CCL2 dramatically reducing this macrophage recruitment [44, 45]. These systemic inflammatory markers may also be driving the higher amount of CD45^+^ immune cells in lactating compared to non‐lactating rats observed 24 h postpartum at the tissue level. Overall, the first 24 h of lactation are instrumental in accelerating PFM regeneration in lactating rats.

The high systemic levels of prolactin and inflammatory markers observed at the start of lactation decrease drastically by 7 days after SBI, to levels lower than those in non‐lactating rats. Compared to lactating rats, 7‐days post‐SBI non‐lactating rats have higher circulating markers of immune cell trafficking, adhesion, and activation, consistent with the expected skeletal muscle temporal immune response to injury [43]. These higher levels of systemic immune cell migration markers are also reflected by the increase in CD45^+^ cells in the pubocaudalis muscle of non‐lactating rats 7‐days after injury. Along with the higher immune cell density, non‐lactating rats have an enrichment in smaller eMyHC^+^ myofibers, indicative of early stages of active repair. Given non‐lactating animals have significantly smaller eMyHC+ myofibers than lactating animals, we evaluated the differentiated muscle stem cell population via MyoG at the 3‐day post‐SBI timepoint as these cells are the source of eMyHC+ myofibers during post‐injury recovery (Figure S6). Lactating animals have a significant increase in MyoG^+^ cells at this early 24‐h postpartum timepoint, leading us to hypothesize that lactating could be accelerating differentiation of muscle stem cells, allowing for rapid remodeling and potential hypertrophy seen long‐term. We also discovered that the high levels of systemic cytokines involved in immune cell adhesion and migration at 1‐week are paralleled in the injured pubocaudalis muscle of non‐lactating rats at the transcriptional level. Overall, the response to birth injury observed at 7 days post‐SBI in non‐lactating animals is present much earlier in the course of muscle recovery in terms of cellular and molecular indices, i.e. at 3 days post‐SBI, in lactating animals. Furthermore, bulk‐RNA‐sequencing revealed that non‐lactating rats have an upregulation of pro‐inflammatory genes when compared to lactating rats that, in contrast, mainly present anti‐inflammatory genes and Tregs at 1‐week. Pubocaudalis muscles of non‐lactating rats undergo enrichment of pathways related to lymphocyte migration and activation, inflammation, and cell adhesion and activation when compared to lactating rats. These genes also support the systemic changes seen in 1‐week postpartum non‐lactating rats, demonstrating an overall heightened immune response. On the other hand, lactating rats at 1‐week have enrichment pathways for myeloid cell development and differentiation, but primarily cell‐adhesion and homeostatic processes, signifying a quicker progression to homeostatic conditions in contrast to the heightened immune activation still seen in non‐lactating rats.

By 3‐weeks postpartum, lactating animals are preparing for weaning and have similar levels of prolactin as non‐lactating rats; however, progesterone remains high at this time point in lactating rats. Progesterone, an endogenous steroid hormone typically produced by the adrenal cortex and ovaries, helps regulate inflammation and stimulate muscle protein synthesis, thus likely aiding in the larger myofibers seen at 3‐weeks after SBI in this group. Despite progesterone remaining high, all circulating cytokines examined show no difference with or without lactation at this time point. This alludes that the systemic immune response decreases by 3‐weeks after injury, regardless of lactation. When assessing morphological differences, larger mature myofibers are present in PFMs of lactating rats, with non‐lactating animals having a higher percentage of small myofibers. These findings illustrate that the larger immature (eMyHC^+^) myofibers seen at 7‐days in lactating rats contribute to larger mature myofibers at 3‐weeks. On a transcriptional level, pubocaudalis muscles in non‐lactating rats continue to be enriched with genes associated with inflammation, cell adhesion, and overall regulation of immune system processes. Notably, PFMs in non‐lactating rats show enrichment of ossification, skeletal system development, and connective tissue development as shown by the higher number of collagens and MMPs, such as *Col19a1, Col15a1, Col5a3*, and *Mmp12*. On the other hand, lactating rats have conserved expression of Tregs and lactation‐specific genes, like *Wap* and *Csn1s2a*, with *Wap* serving as a counter‐regulator of pro‐inflammatory macrophage activation [46]. Overall, by 3‐weeks there are no systemic cytokine differences, but histological and transcriptional changes are still present with non‐lactating rats showing a delay in myofiber maturity and enrichment of pro‐inflammatory genes.

Long‐term outcomes at 4‐ and 8‐weeks after SBI show that the pro‐regenerative phenotype seen throughout lactation persists after weaning of pups. The larger fiber size in lactating rats may be due to the increase in estradiol and testosterone levels that surpass those in non‐lactating and non‐pregnant rats. Testosterone enhances anabolic pathways to increase muscle protein production, stimulates muscle stem cells, and causes muscle hypertrophy [27, 47]. The lack of testosterone in non‐lactating rats may be facilitating an impairment in muscle anabolism to cause atrophy from 4 to 8 weeks. We also assessed collagen content as fibrosis is a well‐established consequence of structural recovery after injury that was previously demonstrated in the non‐pregnant SBI model. Importantly, fibrotic degeneration of PFMs following birth injury does not occur in pregnant rats when compared to uninjured controls, thus differing from the non‐pregnant SBI model [14]. While the pregnant SBI model is characterized by PFM atrophy in non‐lactating rats, it is not a model of fibrotic muscle degeneration, further underscoring the importance for the most translationally relevant preclinical model.

Recent studies have discovered that breastfeeding is associated with reduced maternal risk of cardiovascular diseases and metabolic disorders and is protective against certain cancers. Breastfeeding has also long been associated with calcium deficiency, bone loss, and higher risk of osteoarthritis development [48, 49, 50]. However, recent work has shown that CCN3, a hormone upregulated with lactation, acts as an osteoanabolic factor to build bone in lactating females, leaving uncertainty as to how lactation impacts skeletal muscle injury response [51]. The current study adds to the body of literature supporting benefits of lactation in various contexts. This work also highlights the importance of the postpartum milieu in skeletal muscle regeneration impacted by decreased estrogen levels that are not analogous to widely used estrogen knockout and menopause models. The data suggests that lactation accelerates, but is not required for PFM healing; ultimately, clinical studies will be needed to verify these findings.

There are a few limitations to this study. One is that pup removal may cause other changes beyond lactation removal. While we confirmed that corticosterone levels were not different between groups at 3 days, suggesting no differences in stress levels with pup removal, we cannot fully account for other changes that may have resulted from pup removal. Furthermore, in this work, we assessed structural, cellular, and molecular indices of recovery and performing functional assays will be needed to fully determine functional recovery of these muscles. We also acknowledge that while we were able to assess local immune cell populations and gene expression in the injured pubocaudalis muscles, we did not measure systemic immune cells but rather systemic cytokines and inflammatory factors that could be influencing immune cell mobilization.

Taken together, lactation instills a unique systemic milieu that appears to influence the response of PFMs to birth injury. These findings open many avenues for future studies focused on understanding the mechanisms through which postpartum factors enhance PFM regeneration following maternal birth injury.

## 4 Experimental Section/Methods

### 4.1 Experimental Design

The objective of this study is to assess how lactation impacts the systemic and local injury response of pregnant rats who underwent simulated birth injury. This study was designed to randomly assign rats to non‐lactating (pups removed) and lactating groups to understand the physiological differences that occur with or without breastfeeding.

### 4.2 Animal Model

All procedures were approved by the Institutional Animal Care and Use Committee at the University of California San Diego (IACUC protocol number: S13008). Non‐pregnant and pregnant 3‐month‐old female Sprague‐Dawley rats (Envigo, Indianapolis, IN) were used for all experiments. Pregnant (day 20 gestation) animals underwent SBI and after parturition were randomly divided into non‐lactating (pups removed) and lactating groups. Animals were then euthanized at 3 days and 1, 3, 4, 8, and 12 weeks after SBI was performed. Bilateral pubocaudalis (PCa) muscles and serum were harvested immediately following euthanasia.

### 4.3 Simulated Birth Injury

SBI was performed using a well‐established vaginal balloon distention protocol [12]. Pregnant (Day 20/22) animals were induced with anesthesia at 2.5% isoflurane with oxygen and maintained at 2% for the rest of the procedure. A 16 French silicone‐elastomer coated latex Foley catheter (Medline, Northfield, IL) with the tip cut off was inserted into the vagina and balloon inflated to 10mL. To account for pregnancy‐induced adaptations, we used a 10mL vaginal distention volume which is comparable to the extent of injury previously seen with a 5mL distention volume in non‐pregnant rats [12]. 130 gram weight was attached to the end of the catheter and the balloon was left in place for 2 h. After 2 h, the balloon was deflated to 5mL and pulled through the introitus to simulate the circumferential and downward distention associated with fetal crowning and parturition.

### 4.4 Serum Collection

Blood was collected from each animal by cardiac puncture and remained at room temperature for 30 min before being put on ice. Blood samples were centrifuged at 2000 rcf for 10 min at 4°C. Serum was collected from the supernatant of each sample, aliquoted, and stored at 80°C until use.

### 4.5 Hormone Detection

The Wisconsin National Primate Research Center Assay Services laboratory performed liquid chromatography with triple quadrupole mass spectrometry (LC‐MS/MS) on rat serum for a multi‐analyte analysis of estradiol, estrone, progesterone, testosterone, and corticosterone.

### 4.6 Proteome Profiler Array

A total of 79 proteins were detected with the Proteome Profiler Rat XL Cytokine Array (R&D Systems, Minneapolis, MN) according to the manufacturer's protocol. 700µL of serum was used per membrane. Captured proteins were detected with biotinylated detection antibodies and visualized with 1mL chemiluminescent detection reagent being added to each membrane. The membranes were placed in an autoradiography film cassette and exposed to X‐ray film for 7 min. ImageJ Protein Array Analyzer macro was used to quantify the intensity of positive signals of proteins on all membranes and for normalization to positive reference and negative control signals. Analytes were averaged between duplicates.

### 4.7 Immunohistochemistry

PCa was embedded in OCT, snap‐frozen in isopentane chilled in liquid nitrogen, and stored at ─80°C. PCa was cryosectioned into 10µm cross‐sections and stored at ─80°C until use. Tissue was fixed in acetone for CD45, laminin, embryonic myosin heavy chain (eMyHC), and collagen I stains. For laminin and CD45 staining, slides were washed in PBS, incubated in blocking buffer (5% goat serum, 1% BSA, and 0.05% Triton X‐100 in PBS) for 2 h at room temperature, and incubated with primary antibodies against laminin (1:200, Sigma‐Aldrich, St. Louis, MO, cat#L9393) and CD45 (1:100, BD Biosciences, cat# 554875) in blocking buffer overnight at 4°C. For laminin and eMyHC staining, slides were washed in PBS, incubated with blocking buffer (20% goat serum, 0.3% Triton X‐100 in PBS) for 30 min at room temperature, and incubated with primary antibodies against laminin (1:200, Sigma–Aldrich, St. Louis, MO, cat#L9393), and eMyHC (1:100, DSHB; cat#: F1.652) in blocking buffer overnight at 4°C. The next day, slides were washed with PBS then incubated with secondary antibodies (1:250, Alexa Fluor 647 goat anti‐rabbit IgG and Alexa Fluor 546 goat anti‐mouse IgG) in blocking buffer for 1 h. For collagen I stain, slides were washed in PBS, incubated with blocking buffer (10% goat serum, 1% BSA, 0.3% Triton X‐100 in PBS) for 1 h at room temperature, and then incubated with primary antibody against collagen I (1:200, Bio‐Rad, Hercules, CA, cat#2150‐1908) for 2 h. After washing with PBS, slides were incubated with secondary antibody (1:500, Alexa Fluor 647 goat anti‐rabbit IgG) for 30 min. All slides were incubated with DAPI (1:10 000) for 10 min to identify nuclei and mounted with Fluoromount and a coverslip. Hematoxylin and eosin staining was also performed. Slides were imaged with an Olympus VS200 Slide Scanner.

### 4.8 Immunohistochemistry Analysis

For all immunohistochemistry analyses, investigators were blinded to the group identity.

#### 4.8.1 CD45 Density

For each animal analyzed, a stitched 20x image of the full PCa muscle tissue section near the origin was taken for quantification. QuPath software was used to select CD45+ cells co‐localized with DAPI above a consistent threshold value. The area of the tissue section was taken for normalization.

#### 4.8.2 Myofiber Cross‐Sectional Area

For each animal analyzed, a stitched 20x image of the full PCa muscle tissue section near the origin was taken for quantification. ImageJ custom macro was used for myofiber identification and area measurements.

#### 4.8.3 Embryonic Myosin Heavy Chain

For each PCa, a stitched 20x image of the full PCa muscle tissue section near the origin was used to assess eMyHC+ myofiber cross‐sectional area. ImageJ custom macro was used to determine eMyHC+ myofibers above a consistent threshold for all images.

#### 4.8.4 Collagen I

For each animal analyzed, stitched 20x images of the full PCa muscle tissue sections were used for quantification. Six images were taken throughout the PCa muscle to measure the average collagen I content in the muscle. ImageJ was used to convert cross section images to binary to quantify the percentage of positive collagen I content above a consistent threshold for all images.

### 4.9 Bulk RNA‐Seq Preparation

Pubocaudalis muscles were harvested at 1 and 3 weeks after SBI to assess the expression of genes using bulk RNA sequencing. Muscles were submerged in 1.5mL of RNAlater, stored at 4°C overnight, and then transferred to −80°C until RNA isolation. RNA isolation was performed via a Qiagen RNeasy mini kit following manufacture protocol. RNA concentration was measured with a NanoDrop spectrophotometer and Qubit RNA High Sensitivity equipment. RNA integrity was measured with the Agilent 4200 TapeStation System with manufacture protocol. Pooled samples were then quantified using Qubit 3.0 with a Qubit RNA High Sensitivity kit. Samples were prepped using the Illumina Stranded mRNA kit according to manufacturer protocols and libraries were sequenced at the Institute of Genomic Medicine Genomics Core.

### 4.10 Bulk RNA‐Seq Analysis

A counts matrix was used to input genes into RStudio to be analyzed with an established Bioconductor DESeq2 pipeline with P<0.05 and log2FoldChange>1 cutoffs to assess differentially expressed genes. Differentially expressed genes were then analyzed with Gene Ontology Enrichment Analysis.

### 4.11 Statistical Analysis

Data were analyzed using GraphPad Prism v10, San Diego, CA. P‐values <0.05 were considered significant. For analysis of hormones, a two‐way analysis of variance (ANOVA) was used followed by post hoc pairwise comparisons using Sidak multiple comparison tests. For analysis of systemic immune changes, non‐lactating and lactating animals were compared within each timepoint with two‐way ANOVA, followed by a two‐stage linear step‐up procedure of Benjamini, Krieger and Yekutieli with a false‐discovery rate of 0.05. CD45 cell density was compared with one‐way ANOVA followed by Tukey's multiple comparisons test. Myofiber size distribution was obtained by first eliminating the top and the bottom 1% and subsequently determining the number of bins necessary to describe the distribution (square root of the number of myofibers quantified per biological replicate). To obtain the range for each bin, the bin number was divided by the difference between the maximum and the minimum values. The two conditions were then compared for each bin using two‐way ANOVA followed by post hoc pairwise comparisons using Sidak multiple comparison test. For the analysis of myofiber cross‐sectional area with only two groups, Mann‐Whitney test was performed. For analysis of myofiber cross‐sectional area with more than two groups, Kruskal‐Wallis test was performed followed by Dunn's multiple comparisons test. For the analysis of centralized nuclei with two groups, an unpaired *t*‐test was performed with Welch's correction. For analysis of centralized nuclei with more than two groups, one‐way ANOVA was performed with the Sidak multiple comparison test. For analysis of collagen I content with more than two groups, one‐way ANOVA with Tukey's multiple comparisons test. *p<0.05, **p<0.01, ***p<0.001, ****p<0.0001.

## Author contributions

B.L.P. contributed to the design of experiments, performed in vivo experiments, tissue/blood collection and processing, image analysis, gene expression analysis, and wrote the manuscript. K.M.D., H.K.T, S.C., C.E.L., and L.N.H. performed experiments and analyzed the data. K.L.C. and M.A. contributed to the experimental design, overall project coordination, data interpretation, and writing and editing of the manuscript.

## Conflicts of Interest

The authors declare no conflicts of interest.

## Supporting information




**Supporting File**: advs75945‐sup‐0001‐SuppMat.docx.

## Data Availability

RNA sequencing datasets are openly available in GSE at https://www.ncbi.nlm.nih.gov/geo/query/acc.cgi?acc = GSE328667, reference number 328667. All other data supporting the findings in this study are included in the main article and associated files.

## References

[1] J. M. Wu , C. P. Vaughan , P. S. Goode , et al., “Prevalence and Trends of Symptomatic Pelvic Floor Disorders in U.S. Women,” Obstetrics & Gynecology 123 (2014): 141–148, 10.1097/Aog.0000000000000057. 24463674 PMC3970401

[2] K. A. Kenne , L. Wendt , and J. B. Jackson , “Prevalence of Pelvic Floor Disorders in Adult Women Being Seen in a Primary Care Setting and Associated Risk Factors,” Scientific Reports 12 (2022): 9878, 10.1038/s41598-022-13501-w. 35701486 PMC9198100

[3] J. O. L. DeLancey , M. Masteling , F. Pipitone , J. LaCross , S. Mastrovito , and J. A. Ashton‐Miller , “Pelvic Floor Injury During Vaginal Birth is Life‐Altering and Preventable: What can we do About it?,” American Journal of Obstetrics and Gynecology 230 (2024): 279–294.e2, 10.1016/j.ajog.2023.11.1253. 38168908 PMC11177602

[4] H. U. Memon and V. L. Handa , “Vaginal Childbirth and Pelvic Floor Disorders,” Women's Health 9 (2013): 265–277, 10.2217/whe.13.17. PMC3877300 23638782

[5] T. Sigurdardottir , T. Steingrimsdottir , A. Arnason , and K. Bø , “Pelvic Floor Muscle Function Before and After First Childbirth,” International Urogynecology Journal 22 (2011): 1497–1503, 10.1007/s00192-011-1518-9. 21789656

[6] B. Frohlich , H. Hotzinger , and H. Fritsch , “Tomographical Anatomy of the Pelvis, Pelvic Floor, and Related Structures,” Clinical Anatomy 10 (1997): 223–230. 9213037 10.1002/(SICI)1098-2353(1997)10:4<223::AID-CA1>3.0.CO;2-T

[7] D. J. Jing , J. A. Ashton‐Miller , and J. O. L. DeLancey , “A Subject‐Specific Anisotropic Visco‐Hyperelastic Finite Element Model of Female Pelvic Floor Stress and Strain During the Second Stage of Labor,” Journal of Biomechanics 45 (2012): 455–460, 10.1016/j.jbiomech.2011.12.002. 22209507 PMC3558919

[8] M. Alperin , L. J. Tuttle , B. R. Conner , et al., “Comparison of Pelvic Muscle Architecture Between Humans and Commonly Used Laboratory Species,” International Urogynecology Journal 25 (2014): 1507–1515, 10.1007/s00192-014-2423-9. 24915840 PMC4264598

[9] A. M. Stewart , M. S. Cook , M. C. Esparza , O. D. Slayden , and M. Alperin , “Architectural Assessment of Rhesus Macaque Pelvic Floor Muscles: Comparison for use as a Human Model,” International Urogynecology Journal 28 (2017): 1527–1535, 10.1007/s00192-017-3303-x. 28285397 PMC5593758

[10] F. Fang , Z. Zhao , J. Xiao , J. Wen , J. Wu , and Y. Miao , “Current Practice in Animal Models for Pelvic Floor Dysfunction,” International Urogynecology Journal 34 (2023): 797–808, 10.1007/s00192-022-05387-z. 36287229

[11] V. R. Sheth , P. Duran , J. Wong , et al., “Multimodal Imaging Assessment and Histologic Correlation of the Female Rat Pelvic Floor Muscles″ Anatomy,” Journal of Anatomy 234 (2019): 543–550, 10.1111/joa.12943. 30740685 PMC6422690

[12] T. Catanzarite , S. Bremner , C. L. Barlow , L. Bou‐Malham , S. O'Connor , and M. Alperin , “Pelvic Muscles″ Mechanical Response to Strains in the Absence and Presence of Pregnancy‐Induced Adaptations in a Rat Model,” American Journal of Obstetrics and Gynecology 218 (2018): 512.e1–512.e9, 10.1016/j.ajog.2018.02.001. PMC5916022 29432755

[13] M. Alperin , D. M. Lawley , M. C. Esparza , and R. L. Lieber , “Pregnancy‐Induced Adaptations in the Intrinsic Structure of Rat Pelvic Floor Muscles,” American Journal of Obstetrics and Gynecology 213 (2015): 191.e1–191.e7, 10.1016/j.ajog.2015.05.012. PMC4757427 25979618

[14] P. Duran , F. Boscolo Sesillo , M. Cook , et al., “Proregenerative Extracellular Matrix Hydrogel Mitigates Pathological Alterations of Pelvic Skeletal Muscles After Birth Injury,” Science Translational Medicine 15 (2023): abj3138, 10.1126/scitranslmed.abj3138. PMC10460616 37531414

[15] D. A. Lovejoy , J. L. Roem , J. L. Blomquist , P. R. Pandya , and V. L. Handa , “Breastfeeding and Pelvic Floor Disorders One to Two Decades After Vaginal Delivery,” American Journal of Obstetrics and Gynecology 221 (2019): 333.e1–333.e8, 10.1016/j.ajog.2019.05.010. PMC6766415 31108062

[16] S. Iris , B. Yael , Y. Zehava , et al., “The Impact of Breastfeeding on Pelvic Floor Recovery From Pregnancy and Labor,” European Journal of Obstetrics & Gynecology and Reproductive Biology 251 (2020): 98–105, 10.1016/j.ejogrb.2020.04.017. 32492606

[17] J. L. Voogt , “Control of Hormone Release During Lactation,” Clinics in Obstetrics and Gynaecology 5 (1978): 435–455. 361330

[18] H.‐C. Horng , W.‐H. Chang , C.‐C. Yeh , et al., “Estrogen Effects on Wound Healing,” International Journal of Molecular Sciences 18 (2017): 2325, 10.3390/ijms18112325. 29099810 PMC5713294

[19] R. Chaiyasing , T. Ishikawa , K. Warita , and Y. Z. Hosaka , “Absence of Estrogen Receptors Delays Myoregeneration and Leads to Intermuscular Adipogenesis in a Low Estrogen Status: Morphological Comparisons in Estrogen Receptor Alpha and Beta Knock Out Mice,” Journal of Veterinary Medical Science 83 (2021): 1022–1030, 10.1292/jvms.20-0696. 33967186 PMC8349812

[20] B. C. Collins , R. W. Arpke , A. A. Larson , et al., “Estrogen Regulates the Satellite Cell Compartment in Females,” Cell Reports 28 (2019): 368–381.e6, 10.1016/j.celrep.2019.06.025. 31291574 PMC6655560

[21] S. Xu , F. Xie , L. Tian , et al., “Estrogen Accelerates Heart Regeneration by Promoting the Inflammatory Response in Zebrafish,” Journal of Endocrinology 245 (2020): 39–51, 10.1530/Joe-19-0413. 31977314 PMC7040496

[22] D. Seko , R. Fujita , Y. Kitajima , K. Nakamura , Y. Imai , and Y. Ono , “Estrogen Receptor β Controls Muscle Growth and Regeneration in Young Female Mice,” Stem Cell Reports 15 (2020): 577–586, 10.1016/j.stemcr.2020.07.017. 32822588 PMC7486216

[23] K. E. Knewtson , N. R. Ohl , and J. L. Robinson , “Estrogen Signaling Dictates Musculoskeletal Stem Cell Behavior: Sex Differences in Tissue Repair,” Tissue Engineering Part B: Reviews 28 (2022): 789–812, 10.1089/ten.teb.2021.0094. 34409868 PMC9419932

[24] A. Pellegrino , P. M. Tiidus , and R. Vandenboom , “Mechanisms of Estrogen Influence on Skeletal Muscle: Mass, Regeneration, and Mitochondrial Function,” Sports Medicine 52 (2022): 2853–2869, 10.1007/s40279-022-01733-9. 35907119

[25] F. Boscolo Sesillo , H. Manoochehri , P. Duran , E. Zelus , K. L. Christman , and M. Alperin , “Effect of Lactation on Postpartum Pelvic Floor Muscle Regeneration in Preclinical Model,” NPJ Women's Health 3 (2025): 33, 10.1038/s44294-025-00079-7. 40519212 PMC12162343

[26] S. E. Alexander , A. C. Pollock , and S. Lamon , “The Effect of Sex Hormones on Skeletal Muscle Adaptation in Females,” European Journal of Sport Science 22 (2022): 1035–1045, 10.1080/17461391.2021.1921854. 33890831

[27] O. Horwath , W. Apró , M. Moberg , et al., “Fiber Type‐Specific Hypertrophy and Increased Capillarization in Skeletal Muscle Following Testosterone Administration in Young Women,” Journal of Applied Physiology 128 (2020): 1240–1250, 10.1152/japplphysiol.00893.2019. 32191598

[28] G. I. Smith , J. Yoshino , D. N. Reeds , et al., “Testosterone and Progesterone, But Not Estradiol, Stimulate Muscle Protein Synthesis in Postmenopausal Women,” The Journal of Clinical Endocrinology & Metabolism 99 (2014): 256–265, 10.1210/jc.2013-2835. 24203065 PMC3879672

[29] M. Bekhbat , E. R. Glasper , S. A. Rowson , S. D. Kelly , and G. N. Neigh , “Measuring Corticosterone Concentrations Over a Physiological Dynamic Range in Female Rats,” Physiology & Behavior 194 (2018 May 3): 73–76, 10.1016/j.physbeh.2018.04.033. 29730284 PMC6492035

[30] M. Joëls , H. Karst , and R. A. Sarabdjitsingh , “The Stressed Brain of Humans and Rodents,” Acta Physiologica 223 (2018): 13066, 10.1111/apha.13066. PMC5969253 29575542

[31] H. Ren , P. Yin , and C. Duan , “IGFBP‐5 Regulates Muscle Cell Differentiation by Binding to IGF‐II and Switching on the IGF‐II Auto‐Regulation Loop,” The Journal of Cell Biology 182 (2008): 979–991, 10.1083/jcb.200712110. 18762576 PMC2528583

[32] A. Lu , P. Guo , H. Pan , et al., “Enhancement of Myogenic Potential of Muscle Progenitor Cells and Muscle Healing During Pregnancy,” The FASEB Journal 35 (2021): 21378, 10.1096/fj.202001914r. 33565161

[33] S. Schiaffino , A. C. Rossi , V. Smerdu , L. A. Leinwand , and C. Reggiani , “Developmental Myosins: Expression Patterns and Functional Significance,” Skeletal Muscle 5 (2015): 22, 10.1186/s13395-015-0046-6. 26180627 PMC4502549

[34] N. Dubuisson , R. Versele , C. Planchon , et al., “Histological Methods to Assess Skeletal Muscle Degeneration and Regeneration in Duchenne Muscular Dystrophy,” International Journal of Molecular Sciences 23 (2022): 16080, 10.3390/ijms232416080. 36555721 PMC9786356

[35] T. S. Wilkinson , A. Roghanian , A. J. Simpson , and J.‐M. Sallenave , “WAP Domain Proteins as Modulators of Mucosal Immunity,” Biochemical Society Transactions 39 (2011): 1409–1415, 10.1042/bst0391409. 21936824

[36] A. M. Stuebe and J. W. Rich‐Edwards , “The Reset Hypothesis: Lactation and Maternal Metabolism,” American Journal of Perinatology 26 (2009): 081–088, 10.1055/s-0028-1103034. PMC3006166 19031350

[37] K. Yoh , K. Ikeda , K. Horie , and S. Inoue , “Roles of Estrogen, Estrogen Receptors, and Estrogen‐Related Receptors in Skeletal Muscle: Regulation of Mitochondrial Function,” International Journal of Molecular Sciences 24 (2023): 1853, 10.3390/ijms24031853. 36768177 PMC9916347

[38] S. R. Valdez , A. B. Penissi , R. P. Deis , and G. A. Jahn , “Hormonal Profile and Reproductive Performance in Lactation Deficient (OFA hr/hr) and Normal (Sprague–Dawley) Female Rats,” Reproduction 133 (2007): 827–840, 10.1530/rep-06-0032. 17504926

[39] M. E. Freeman , B. Kanyicska , A. Lerant , and G. Nagy , “Prolactin: Structure, Function, and Regulation of Secretion,” Physiological Reviews 80 (2000): 1523–1631, 10.1152/physrev.2000.80.4.1523. 11015620

[40] I. Medina‐Estrada , N. Alva‐Murillo , J. E. López‐Meza , and A. Ochoa‐Zarzosa , “Non‐Classical Effects of Prolactin on the Innate Immune Response of Bovine Mammary Epithelial Cells: Implications During Staphylococcus Aureus Internalization,” Microbial Pathogenesis 89 (2015): 43–53, 10.1016/j.micpath.2015.08.018. 26341952

[41] M. Rapp , M. W. M. Wintergerst , et al., W. G. Kunz , “CCL22 Controls Immunity by Promoting Regulatory T Cell Communication With Dendritic Cells in Lymph Nodes,” Journal of Experimental Medicine 216 (2019): 1170–1181, 10.1084/jem.20170277. 30910796 PMC6504218

[42] A. Strömberg , E. Rullman , E. Jansson , and T. Gustafsson , “Exercise‐Induced Upregulation of Endothelial Adhesion Molecules in Human Skeletal Muscle and Number of Circulating Cells With Remodeling Properties,” Journal of Applied Physiology 122 (2017): 1145–1154, 10.1152/japplphysiol.00956.2016. 28183821

[43] J. G. Tidball , “Regulation of Muscle Growth and Regeneration by the Immune System,” Nature Reviews Immunology 17 (2017): 165–178, 10.1038/nri.2016.150. PMC5452982 28163303

[44] H. Lu , D. Huang , R. M. Ransohoff , and L. Zhou , “Acute Skeletal Muscle Injury: CCL2 Expression by Both Monocytes and Injured Muscle is Required for Repair,” The FASEB Journal 25: 3344–3355, 10.1096/fj.10-178939. PMC3177578 21697550

[45] P. J. Ferrara , P. T. Reidy , J. J. Petrocelli , et al., “Global Deletion of CCL2 has Adverse Impacts on Recovery of Skeletal Muscle Fiber Size and Function and is Muscle Specific,” Journal of Applied Physiology 134 (2023): 923–932, 10.1152/japplphysiol.00444.2022. 36861669 PMC10069960

[46] M. Karlstetter , Y. Walczak , K. Weigelt , et al., “The Novel Activated Microglia/Macrophage WAP Domain Protein, AMWAP, Acts as a Counter‐Regulator of Proinflammatory Response,” The Journal of Immunology 185 (2010): 3379–3390, 10.4049/jimmunol.0903300. 20709948

[47] J. Tapper , G. Huang , K. M. Pencina , et al., “The Effects of Testosterone Administration on Muscle Areas of the Trunk and Pelvic Floor in Hysterectomized Women With Low Testosterone Levels: Proof‐of‐Concept Study,” Menopause 26 (2019): 1405–1414, 10.1097/GME.0000000000001410. 31479032 PMC6893124

[48] P. Affinito , G. A. Tommaselli , C. Di Carlo , F. Guida , and C. Nappi , “Changes in Bone Mineral Density and Calcium Metabolism in Breastfeeding Women: A One Year Follow‐Up Study,” The Journal of Clinical Endocrinology & Metabolism 81 (1996): 2314–2318, 10.1210/jcem.81.6.8964870. 8964870

[49] C. Y. Park , “Breastfeeding for One Month or Longer is Associated With Higher Risk of Osteoarthritis in Older Adults: NHANES 1999–2012,” Clinical Nutrition Research 6 (2017): 277–284, 10.7762/cnr.2017.6.4.277. 29124048 PMC5665749

[50] F. M. F. Grizzo , A. C. J. Alarcão , et al., C. M. Dell' Agnolo , “How Does Women's Bone Health Recover After Lactation? A Systematic Review and Meta‐Analysis,” Osteoporosis International 31 (2020): 413–427, 10.1007/s00198-019-05236-8. 31897544

[51] M. E. Babey , W. C. Krause , K. Chen , et al., “A Maternal Brain Hormone That Builds Bone,” Nature 632 (2024): 357–365, 10.1038/s41586-024-07634-3. 38987585 PMC11306098

