# Supplementary material for: Lactation is Associated with Accelerated Postpartum Pelvic Floor Muscle Recovery in a Pregnant Simulated Birth Injury Model
Source: Adv Sci (Weinh). 2026 Jun 9;13(49):e00052. doi: 10.1002/advs.202600052 (PMC13336909; doi:10.1002/advs.202600052)
Supplement: Supplementary file 1 [file ADVS-13-e00052-s001.docx]

**Supporting Information**

Lactation is associated with accelerated postpartum pelvic floor muscle recovery in a pregnant simulated birth injury model

*Bianca L. Peña^1,2^, Khushi M. Diwakar^2,3^, Hillary K. Tran^1,2^, Selena Cao^1,2^, Celeste E. Lintz^1,2^, Laila N. Hayes^1,2^, Marianna Alperin^2,4^*, Karen L. Christman^1,2,5^*

*Co-corresponding Authors

**Affiliations**

1. Shu Chien-Gene Lay Department of Bioengineering, University of California, San Diego, La Jolla, CA, 92093, USA
2. Sanford Consortium for Regenerative Medicine, La Jolla, CA, 92037, USA
3. School of Biological Sciences, University of California San Diego, La Jolla, CA, 92093, USA
4. Department of Obstetrics, Gynecology, and Reproductive Sciences, Division of Urogynecology and Reconstructive Pelvic Surgery, University of California San Diego, La Jolla, CA 92093, USA
5. Sanford Stem Cell Institute, La Jolla, CA 92037

**Supplemental Figure 1.** Corticosterone serum levels in non-lactating and lactating rats at 3-days after SBI, 24 hours postpartum. Mean ±SEM. Non-pregnant values shown as mean ±SEM in grey. 3d SBI (n=6), 3d SBI +Lac (n=5). Unpaired t-test.

**Supplemental Figure 2.** Systemic cytokine profile of non-lactating and lactating rats 3-days after SBI, 1-day postpartum. **(A)** Tissue remodeling and extracellular matrix proteins. **(B)** Multifunctional proteins. **(C)** Growth factors. **(D)** Soluble receptors and adhesion molecules. **(E)** Cytokines. **(F)** Adipokines and metabolic regulators. **(G)** Chemokines.

**Supplemental Figure 3.** Systemic cytokine profile of non-lactating and lactating rats 1-week after SBI. **(A)** Tissue remodeling and extracellular matrix proteins. **(B)** Multifunctional proteins. **(C)** Growth factors. **(D)** Soluble receptors and adhesion molecules. **(E)** Cytokines. **(F)** Adipokines and metabolic regulators. **(G)** Chemokines.

**Supplemental Figure 4.** Systemic cytokine profile of non-lactating and lactating rats 3-weeks after SBI. **(A)** Tissue remodeling and extracellular matrix proteins. **(B)** Multifunctional proteins. **(C)** Growth factors. **(D)** Soluble receptors and adhesion molecules. **(E)** Cytokines. **(F)** Adipokines and metabolic regulators. **(G)** Chemokines.

**Supplemental Figure 5.** Macrophage populations were not significantly different between groups at 7-days post-SBI. **(A)** Bar graph representing quantification of CD68+ cells and **(B)** CD163+ cells normalized to tissue section area via immunohistochemistry. 7d SBI (N=5), 7d SBI+Lac (N=5). Unpaired two-tailed t-test.

**Supplemental Figure 6.** The pubocaudalis muscle of lactating animals have more MyoG+ cells at 3-days after SBI. **(A)** Immunofluorescent staining against laminin (red), myogenin (MyoG, green), and DAPI (blue) in pubocaudalis muscle cross sections in non-lactating and lactating animals. **(B)** Bar graph representing quantification of MyoG+ cells normalized to tissue section area. Scale bars are 100µm, 3d SBI (N=6), 3d SBI+Lac (N=5). Unpaired two-tailed t-test, *P<0.05.

**Table S1.** List of differentially expressed genes for bulk RNA sequencing transcriptomics experiment, 1-week post-SBI non-lactating vs 1-week post-SBI lactating. Genes listed are differentially expressed in 1-week post-SBI non-lactating animals.

| **Gene** | **Log2 Fold Change** | **padj** |
| --- | --- | --- |
| Prrg4 | -1.00018 | 6.00E-06 |
| Spon1 | -1.00592 | 0.006594 |
| Camk1d | -1.00946 | 0.004244 |
| Cd83 | -1.01096 | 0.000294 |
| P2ry12 | -1.01258 | 0.000643 |
| Sh3bp2 | -1.01443 | 0.000196 |
| Ptn | -1.018 | 1.06E-05 |
| Galc | -1.01881 | 0.001255 |
| Thbs1 | -1.02113 | 0.000412 |
| F5 | -1.02127 | 0.024078 |
| Aldh1l2 | -1.02446 | 0.010643 |
| Tent5c | -1.02452 | 0.002902 |
| P2rx4 | -1.02619 | 1.58E-06 |
| Pax7 | -1.02748 | 0.011221 |
| Adamts14 | -1.02785 | 0.006636 |
| Gna15 | -1.0291 | 0.019258 |
| Btk | -1.03584 | 0.041562 |
| Mycl | -1.03967 | 0.044395 |
| Nt5dc2 | -1.04002 | 0.01166 |
| Aoah | -1.04444 | 0.001454 |
| Diaph3 | -1.04527 | 0.040015 |
| Abcg1 | -1.04866 | 1.70E-08 |
| Vav1 | -1.05466 | 7.46E-05 |
| Stx3 | -1.05522 | 0.001194 |
| Cd109 | -1.05593 | 0.019521 |
| Chst11 | -1.05604 | 0.035536 |
| Sdc1 | -1.06022 | 1.77E-05 |
| Itgb2 | -1.06235 | 0.000107 |
| Sec1 | -1.06406 | 0.030963 |
| Capn6 | -1.06602 | 0.000248 |
| Cd33 | -1.07412 | 0.005513 |
| Lrch2 | -1.07447 | 0.027851 |
| Otulinl | -1.07567 | 0.005916 |
| Dpep2 | -1.07966 | 0.008972 |
| Cthrc1 | -1.0852 | 0.000138 |
| Kcnk13 | -1.0875 | 0.032922 |
| Septin6 | -1.09175 | 0.000385 |
| Ms4a6c | -1.09627 | 0.015905 |
| Ccdc141 | -1.10118 | 0.000483 |
| C3ar1 | -1.10528 | 5.36E-05 |
| Vcam1 | -1.10532 | 1.29E-06 |
| Rgs18 | -1.10542 | 0.000246 |
| C1h10orf143 | -1.10587 | 0.024031 |
| Nfkbiz | -1.10592 | 0.012623 |
| Tmem86a | -1.10844 | 5.45E-05 |
| Cd28 | -1.10962 | 0.011046 |
| Ror2 | -1.10979 | 0.014546 |
| Zbtb7c | -1.10981 | 0.006649 |
| Samsn1 | -1.11283 | 0.022347 |
| Sytl2 | -1.11311 | 1.77E-05 |
| Hvcn1 | -1.11515 | 1.40E-05 |
| Wt1 | -1.11768 | 0.025691 |
| Acap1 | -1.12042 | 0.021378 |
| Top2a | -1.12571 | 0.032123 |
| Erbb3 | -1.1365 | 0.010532 |
| Sla | -1.13924 | 0.005361 |
| Megf10 | -1.13972 | 1.14E-05 |
| Dcx | -1.14216 | 0.003629 |
| Fbxw9 | -1.14827 | 0.027567 |
| Igsf6 | -1.15309 | 0.000784 |
| Sqle | -1.15739 | 0.011953 |
| Cd180 | -1.17236 | 0.001432 |
| Apoe | -1.17337 | 1.27E-07 |
| Racgap1 | -1.18223 | 0.043787 |
| Fgr | -1.18611 | 0.000113 |
| Mest | -1.18915 | 0.002835 |
| P4ha3 | -1.19669 | 0.002903 |
| Slc39a8 | -1.20087 | 0.01 |
| Clec7a | -1.20913 | 0.000176 |
| Htr7 | -1.21051 | 0.032468 |
| Ccdc159 | -1.21136 | 0.027851 |
| Anln | -1.21157 | 0.004101 |
| Fgd3 | -1.21261 | 0.032281 |
| Skp2 | -1.21579 | 0.030741 |
| Slamf7 | -1.22539 | 0.009193 |
| Gpr34 | -1.22698 | 0.001352 |
| Shisal1 | -1.22759 | 0.022189 |
| Zdbf2 | -1.23009 | 0.001655 |
| Clec4a | -1.23011 | 0.003509 |
| Vipr2 | -1.23058 | 0.033487 |
| Cd84 | -1.23126 | 0.000204 |
| Map7 | -1.23922 | 0.009296 |
| Scd | -1.24697 | 5.80E-05 |
| Fbn2 | -1.25369 | 0.015579 |
| Psd4 | -1.2547 | 0.002903 |
| Sell | -1.25867 | 0.049047 |
| Tubb2a | -1.26299 | 0.00015 |
| Asns | -1.26746 | 0.014841 |
| Cpa1 | -1.26808 | 0.000496 |
| Slamf9 | -1.27237 | 5.49E-05 |
| Neto2 | -1.27295 | 0.04597 |
| Nes | -1.27681 | 6.50E-05 |
| Cpxm1 | -1.2779 | 0.000364 |
| C1qtnf3 | -1.28469 | 9.72E-05 |
| Lmnb2 | -1.28659 | 4.48E-05 |
| Ccne2 | -1.29127 | 0.001428 |
| Itgax | -1.29468 | 0.032931 |
| Ptrh1 | -1.29501 | 0.005829 |
| Armcx2 | -1.30398 | 0.000721 |
| Ptprn | -1.30675 | 0.014866 |
| Abca17 | -1.30886 | 0.010803 |
| Edar | -1.3152 | 0.032387 |
| S100a4 | -1.31924 | 3.28E-05 |
| Blnk | -1.32454 | 1.22E-05 |
| Kif23 | -1.32916 | 0.030647 |
| Prc1 | -1.33947 | 0.002712 |
| Rbm47 | -1.35257 | 7.30E-05 |
| Dok3 | -1.3535 | 0.000918 |
| LOC502907 | -1.35828 | 1.27E-07 |
| Kif20a | -1.36902 | 0.030583 |
| Map6d1 | -1.36923 | 0.004893 |
| Crabp2 | -1.36976 | 0.006636 |
| Mcm5 | -1.37483 | 0.004415 |
| Mpeg1 | -1.38644 | 1.91E-08 |
| Rbm3 | -1.39206 | 1.71E-17 |
| LOC24906 | -1.39277 | 0.012409 |
| Gcsam | -1.39344 | 0.009566 |
| Actc1 | -1.40242 | 0.018517 |
| Slc15a3 | -1.40632 | 6.50E-05 |
| Irf8 | -1.40641 | 1.03E-09 |
| Cnr2 | -1.41064 | 0.020699 |
| LOC691141 | -1.4142 | 0.003114 |
| Runx2 | -1.41491 | 0.004023 |
| Bub1b | -1.41687 | 0.01524 |
| Adgrg5 | -1.4272 | 0.032124 |
| Sox9 | -1.42892 | 0.006143 |
| Iqgap3 | -1.43562 | 0.000918 |
| Lrrc15 | -1.4385 | 0.002312 |
| RGD1563400 | -1.43974 | 0.027049 |
| Zfand4 | -1.44558 | 0.005586 |
| Fam171a2 | -1.4531 | 0.000119 |
| Cx3cr1 | -1.45419 | 0.042688 |
| Cdca7 | -1.46103 | 0.008399 |
| Dnah1 | -1.46853 | 0.037957 |
| Mki67 | -1.47272 | 6.63E-05 |
| Cenpf | -1.48056 | 0.021251 |
| Col11a1 | -1.49655 | 8.33E-09 |
| Ccn4 | -1.49685 | 5.10E-08 |
| Ptk7 | -1.50524 | 1.44E-05 |
| Arl4c | -1.50594 | 1.13E-05 |
| Nrk | -1.50719 | 0.01616 |
| Cdk5r1 | -1.51328 | 6.00E-06 |
| Sox11 | -1.51427 | 0.025791 |
| Ly49si1 | -1.51824 | 3.13E-14 |
| Ect2 | -1.52165 | 0.033366 |
| Nrcam | -1.52655 | 0.006412 |
| Tnc | -1.52803 | 0.029308 |
| Tmem119 | -1.53174 | 1.30E-05 |
| Krt8 | -1.55377 | 0.048656 |
| Cdca3 | -1.56091 | 0.00859 |
| Epha1 | -1.56268 | 0.021728 |
| Spib | -1.56829 | 0.016681 |
| Btla | -1.57741 | 0.017444 |
| Ptprv | -1.58103 | 0.007306 |
| Myl4 | -1.58612 | 0.019775 |
| Itgal | -1.58982 | 1.29E-06 |
| Tgif2 | -1.5926 | 0.001556 |
| Pkp1 | -1.59994 | 0.001697 |
| Gapt | -1.60117 | 0.004274 |
| Peg12 | -1.60182 | 0.028527 |
| Mdk | -1.60496 | 0.000723 |
| Depdc7 | -1.61145 | 0.012256 |
| LOC497796 | -1.61414 | 0.000286 |
| Kif4a | -1.61531 | 0.002103 |
| Gpnmb | -1.61568 | 4.36E-13 |
| Fcrl2 | -1.62053 | 2.95E-05 |
| Parvg | -1.62124 | 0.030939 |
| Lilrb4 | -1.62431 | 2.26E-05 |
| Tac3 | -1.62769 | 0.003629 |
| Myo16 | -1.63015 | 0.011644 |
| Bard1 | -1.65068 | 0.041098 |
| Mex3a | -1.65612 | 0.011644 |
| Ly75 | -1.6581 | 0.026254 |
| Sele | -1.66017 | 0.026794 |
| Cdh2 | -1.66293 | 0.000106 |
| Kcnj15 | -1.66341 | 0.000301 |
| Cacna1d | -1.66733 | 0.032387 |
| Adam12 | -1.67946 | 0.009751 |
| Cdk1 | -1.68239 | 0.004101 |
| Syn1 | -1.68412 | 0.043197 |
| B4galnt1 | -1.68872 | 0.04687 |
| Il1rn | -1.69591 | 0.022994 |
| Jaml | -1.69642 | 4.53E-05 |
| Jchain | -1.69742 | 6.89E-05 |
| Clec9a | -1.70643 | 0.004963 |
| Kif11 | -1.70756 | 0.001118 |
| Plk4 | -1.70829 | 0.001713 |
| Tlr1 | -1.71865 | 0.013139 |
| E2f8 | -1.73655 | 0.001656 |
| Mymk | -1.74203 | 0.03359 |
| Faah | -1.74221 | 0.000236 |
| Fndc3c1 | -1.74392 | 6.72E-06 |
| Gabbr2 | -1.75357 | 0.002596 |
| Slpi | -1.76154 | 0.000196 |
| Ddn | -1.76284 | 0.000733 |
| Cldn2 | -1.77118 | 0.002057 |
| A2m | -1.77769 | 0.000359 |
| Tubb2b | -1.78001 | 3.99E-07 |
| Frmpd1 | -1.78898 | 3.99E-06 |
| Gcgr | -1.81466 | 0.007432 |
| Dclk1 | -1.82058 | 0.035696 |
| Klra5 | -1.82446 | 0.036785 |
| Baiap2l1 | -1.8252 | 0.000132 |
| Cxcr3 | -1.84683 | 0.036606 |
| St8sia2 | -1.85382 | 9.88E-05 |
| S100a3 | -1.86272 | 0.000945 |
| Vwa2 | -1.8659 | 0.020119 |
| Grem1 | -1.89547 | 0.008289 |
| Tmem178a | -1.89831 | 0.000301 |
| Col6a5 | -1.90503 | 0.005623 |
| Clec2dl1 | -1.91393 | 0.002057 |
| Tmem200a | -1.91634 | 0.007487 |
| Lilrb2 | -1.9202 | 9.29E-05 |
| Kmo | -1.92187 | 0.030083 |
| Cimap1c | -1.95226 | 0.001099 |
| Draxin | -1.95513 | 0.000365 |
| Ttk | -1.96991 | 0.006269 |
| Myh8 | -1.97743 | 0.024011 |
| Dbf4 | -1.98159 | 0.000537 |
| LOC102547811 | -2.00166 | 0.011644 |
| Sox8 | -2.00211 | 0.000496 |
| Ckap2l | -2.02491 | 0.015465 |
| LOC102549548 | -2.04141 | 0.000348 |
| Cd8b | -2.04503 | 0.010616 |
| Dtl | -2.06328 | 0.019552 |
| Siglec10 | -2.09762 | 0.002443 |
| Ncaph | -2.15234 | 0.002312 |
| Megf6 | -2.1525 | 2.75E-05 |
| Klrc3 | -2.16727 | 0.043675 |
| Ccr7 | -2.17237 | 0.027002 |
| Kif26b | -2.20653 | 0.020119 |
| Ccr9 | -2.21222 | 0.009843 |
| Dlg2 | -2.21337 | 0.028719 |
| Folr1 | -2.25536 | 0.017529 |
| Or4e5 | -2.27469 | 0.01524 |
| Podnl1 | -2.3264 | 2.44E-05 |
| LOC685989 | -2.33281 | 0.007643 |
| Prss35 | -2.33892 | 2.51E-05 |
| Reep2 | -2.3431 | 0.018194 |
| Cenph | -2.40738 | 0.027851 |
| Bend5 | -2.41769 | 0.047649 |
| Siglec15 | -2.47858 | 0.037544 |
| Col26a1 | -2.49278 | 7.59E-05 |
| Galnt6 | -2.52873 | 0.048833 |
| Rassf7 | -2.54164 | 0.030939 |
| B3gnt5 | -2.55562 | 0.005184 |
| Plekhg4 | -2.55672 | 0.000356 |
| Clec4a2 | -2.68811 | 0.014888 |
| Cd5l | -2.69785 | 0.026791 |
| Phex | -2.77038 | 0.022937 |
| Cyp11b1 | -2.9109 | 0.004144 |
| Rnase3 | -2.91163 | 0.018418 |
| Shc3 | -2.94437 | 0.008459 |
| Spp1 | -3.01465 | 4.31E-23 |
| Bicdl2 | -3.08494 | 0.038027 |
| Klrd1 | -3.18159 | 0.002631 |
| Tnn | -3.18754 | 0.002134 |
| Slc44a3 | -3.32012 | 8.23E-05 |
| Sirpb3 | -3.54725 | 0.027326 |
| LOC103690642 | -3.77459 | 6.96E-05 |
| Mmp9 | -3.79105 | 0.002431 |
| Acan | -4.13935 | 0.001151 |
| Foxm1 | -4.35969 | 0.01199 |
| Mmp12 | -4.6724 | 0.000366 |
| Slc4a5 | -4.71391 | 0.003709 |
| Casr | -4.81065 | 0.024887 |
| Adamts18 | -4.87908 | 0.022448 |
| Bicdl1 | -4.9557 | 0.01182 |
| Dnah17 | -4.99005 | 0.010446 |

**Table S2.** List of differentially expressed genes for bulk RNA sequencing transcriptomics experiment, 1-week post-SBI non-lactating vs 1-week post-SBI lactating. Genes listed are differentially expressed in 1-week post-SBI lactating animals.

| **Gene** | **log2FoldChange** | **padj** |
| --- | --- | --- |
| Csn2 | 8.010851 | 2.38E-06 |
| Csn1s1 | 7.92013 | 1.13E-05 |
| Zfp300 | 7.686208 | 7.28E-06 |
| NA | 7.464417 | 1.44E-05 |
| Hpd | 4.326869 | 0.012577 |
| Lrrc69 | 3.706202 | 0.034584 |
| LOC678760 | 2.33675 | 0.04634 |
| Inca1 | 2.332065 | 0.035696 |
| Dipk1c | 2.259263 | 0.01645 |
| NA | 2.148676 | 5.39E-15 |
| Mc5r | 1.974048 | 5.32E-06 |
| Oas1k | 1.818716 | 0.046352 |
| Ces1a | 1.742377 | 0.030228 |
| Cabp1 | 1.739263 | 0.026942 |
| Slc4a1 | 1.722535 | 0.011985 |
| Ift70a2 | 1.628244 | 0.04436 |
| Angptl4 | 1.587812 | 0.00417 |
| Alox15 | 1.548816 | 0.009576 |
| Ntsr1 | 1.470144 | 0.018344 |
| Hist1h2ao | 1.445331 | 7.95E-07 |
| Fkbp5 | 1.416681 | 2.65E-07 |
| Ptpru | 1.365222 | 0.040033 |
| Fmo2 | 1.325091 | 1.24E-05 |
| Sult1a1 | 1.302322 | 3.78E-05 |
| Alas2 | 1.298759 | 0.006348 |
| Slc15a2 | 1.289431 | 2.97E-05 |
| Lvrn | 1.235966 | 1.89E-05 |
| Hba-a1 | 1.219394 | 4.08E-06 |
| Lrrc66 | 1.184978 | 0.042817 |
| Asb11 | 1.172558 | 1.15E-05 |
| Glul | 1.146152 | 3.38E-07 |
| Hbb | 1.110282 | 2.21E-05 |
| Prss36 | 1.104987 | 0.03133 |
| Il15 | 1.102163 | 0.001127 |
| Cntfr | 1.097614 | 0.005513 |
| Bmp7 | 1.090619 | 0.049715 |
| Lmod1 | 1.086462 | 0.001654 |
| Dusp26 | 1.086403 | 3.38E-05 |
| Sncg | 1.064165 | 3.06E-05 |
| Asb18 | 1.034548 | 0.011644 |
| Sqor | 1.028686 | 0.000348 |
| Nfil3 | 1.028208 | 9.35E-11 |
| Zbtb16 | 1.016713 | 0.001891 |
| Ppp1r1a | 1.015924 | 5.24E-06 |
| Inmt | 1.000444 | 0.030375 |

**Table S3.** List of differentially expressed genes for bulk RNA sequencing transcriptomics experiment, 3-week post-SBI non-lactating vs 3-week post-SBI lactating. Genes listed are differentially expressed in 3-week post-SBI non-lactating animals.

| **Gene** | **log2FoldChange** | **padj** |
| --- | --- | --- |
| Dmxl2 | -1.011019302 | 0.000151 |
| Scarb1 | -1.017979824 | 0.000319 |
| NA | -1.025322773 | 0.003792 |
| Ndrg4 | -1.037765709 | 0.011855 |
| Olfm1 | -1.037837991 | 0.006793 |
| Lgals3bp | -1.038096325 | 0.000238 |
| Hcls1 | -1.039825754 | 0.027465 |
| NA | -1.041589155 | 0.015238 |
| Psmb8 | -1.06032803 | 0.048073 |
| Thbs3 | -1.064003631 | 0.019733 |
| Col11a1 | -1.067887326 | 0.048851 |
| Fam171a2 | -1.068858694 | 0.028649 |
| Col15a1 | -1.075154927 | 1.36E-08 |
| Mlkl | -1.077350366 | 0.018031 |
| Rbm3 | -1.082337764 | 3.16E-09 |
| Cd68 | -1.083440631 | 0.000275 |
| Gstt3 | -1.090024488 | 0.032511 |
| Fap | -1.094797194 | 0.003273 |
| S100a4 | -1.095091996 | 0.012512 |
| Ucp2 | -1.098738453 | 0.002036 |
| Col5a3 | -1.099436171 | 0.000347 |
| Fst | -1.100607341 | 0.003815 |
| Adamts12 | -1.106340549 | 0.002958 |
| Cdkn1c | -1.10908275 | 0.001508 |
| Cilp | -1.11838053 | 3.61E-05 |
| Dok5 | -1.129935935 | 0.018336 |
| Npnt | -1.131199097 | 1.62E-06 |
| Rin1 | -1.156833385 | 0.045249 |
| NA | -1.162846932 | 0.033572 |
| Prrg4 | -1.166710339 | 0.035943 |
| Igsf1 | -1.167395318 | 0.034656 |
| C3ar1 | -1.182993191 | 0.024158 |
| Coro1a | -1.18455404 | 0.049579 |
| Hr | -1.184848108 | 0.001964 |
| Stmn2 | -1.19724482 | 0.013959 |
| Lhfpl2 | -1.202545094 | 0.004888 |
| Cdkn1a | -1.205754961 | 0.038396 |
| Megf10 | -1.217888507 | 0.012084 |
| Scube2 | -1.21820698 | 0.018933 |
| Col6a6 | -1.230807555 | 0.001022 |
| Dkk3 | -1.231907851 | 0.00164 |
| Pax7 | -1.234285823 | 0.013544 |
| Mt1 | -1.238320863 | 0.047882 |
| Gbp1 | -1.244499677 | 0.001081 |
| Tnfrsf19 | -1.260951951 | 0.027191 |
| Kcne5 | -1.283850737 | 0.030913 |
| Mill1 | -1.285708851 | 0.008822 |
| Nnat | -1.28648144 | 0.037551 |
| Klk1 | -1.295762419 | 0.001401 |
| Dnah5 | -1.302834328 | 0.020253 |
| Micall2 | -1.304320972 | 0.039924 |
| Mx1 | -1.30595658 | 0.005687 |
| Parm1 | -1.308467193 | 5.27E-07 |
| Irf7 | -1.309391388 | 6.71E-07 |
| Tmem158 | -1.310756884 | 0.018361 |
| Adam19 | -1.31135801 | 1.34E-07 |
| Foxred2 | -1.338649429 | 0.022029 |
| Gpnmb | -1.344607092 | 9.18E-10 |
| Cpxm1 | -1.345736062 | 0.00222 |
| Rrad | -1.349307528 | 3.73E-05 |
| NA | -1.350779593 | 0.034232 |
| Rarres1 | -1.37271451 | 0.003481 |
| Cpt1a | -1.378960568 | 2.09E-06 |
| Scn5a | -1.407786561 | 0.032358 |
| Adamts7 | -1.411842667 | 0.031855 |
| Siglec1 | -1.42090126 | 0.000676 |
| Frmpd1 | -1.453792215 | 0.003096 |
| Sncg | -1.456695295 | 1.28E-11 |
| Slamf9 | -1.461197814 | 0.021166 |
| Mgmt | -1.467202542 | 0.000509 |
| Sec1 | -1.477289229 | 0.038645 |
| C1qtnf6 | -1.487036581 | 0.002546 |
| Rps15al4 | -1.495079647 | 0.002085 |
| Maged2 | -1.50192173 | 2.59E-10 |
| Slc30a2 | -1.510536651 | 0.047523 |
| Zp2 | -1.511169337 | 0.008453 |
| Igsf10 | -1.531091068 | 4.67E-05 |
| Btnl9 | -1.542349553 | 0.010869 |
| Prr32 | -1.543493732 | 0.000143 |
| Flt3 | -1.54842161 | 0.018426 |
| Fndc3c1 | -1.553095518 | 0.004286 |
| Pcdh17 | -1.604189222 | 0.027612 |
| Dnase1l3 | -1.611472233 | 0.003297 |
| Papln | -1.62164348 | 3.04E-05 |
| Flywch2 | -1.626840511 | 0.001126 |
| Glrx | -1.627139907 | 5.75E-06 |
| St8sia2 | -1.635159446 | 0.039924 |
| Mest | -1.645449947 | 1.04E-06 |
| Lilrb3 | -1.665574117 | 0.046822 |
| Ankrd29 | -1.669567873 | 0.042454 |
| Jaml | -1.669823252 | 0.019867 |
| Mx2 | -1.702203703 | 6.71E-07 |
| Cd180 | -1.718265026 | 0.047211 |
| Sema3a | -1.747738184 | 0.000118 |
| Ostn | -1.748071798 | 0.047744 |
| RGD1562029 | -1.761304974 | 0.020842 |
| Myh15 | -1.762123356 | 0.00021 |
| Mycl | -1.764205991 | 0.030702 |
| Ppp1r14c | -1.798211824 | 1.32E-06 |
| Spp1 | -1.805560147 | 0.000348 |
| Kcnj3 | -1.841121147 | 0.035126 |
| Rsad2 | -1.849311694 | 0.024939 |
| Nmrk2 | -1.850240874 | 0.018745 |
| Igfn1 | -1.854524316 | 8.99E-10 |
| Prrt4 | -1.863016106 | 0.000607 |
| NA | -1.871146324 | 0.000103 |
| Emb | -1.873055724 | 1.71E-06 |
| Adam12 | -1.897671738 | 0.001958 |
| Aldh1l2 | -1.949236213 | 0.023901 |
| Cd28 | -1.991900676 | 3.49E-08 |
| Faah | -2.026645264 | 0.015236 |
| Col19a1 | -2.05429159 | 3.48E-18 |
| Car12 | -2.074643259 | 0.000676 |
| NA | -2.100212275 | 0.009516 |
| Sytl1 | -2.111792252 | 0.020729 |
| NA | -2.113733486 | 3.44E-05 |
| Scd | -2.116522806 | 0.000357 |
| Prc1 | -2.133356015 | 0.001456 |
| NA | -2.138242605 | 0.029513 |
| Cenpf | -2.21908039 | 0.026583 |
| Spmip6 | -2.244366139 | 0.027964 |
| Col6a5 | -2.290072357 | 0.017475 |
| Peg10 | -2.299782351 | 3.57E-06 |
| Tigd4 | -2.464901211 | 0.019896 |
| NA | -2.62514811 | 0.000473 |
| Siglec10 | -2.643217829 | 0.000227 |
| NA | -2.646653694 | 0.029938 |
| Myo5b | -2.686653927 | 0.049007 |
| Angptl4 | -2.725526099 | 0.000151 |
| Anxa8 | -3.055621741 | 2.17E-10 |
| Prnd | -3.243439179 | 3.96E-05 |
| Mmp12 | -5.821565004 | 3.96E-05 |

**Table S4.** List of differentially expressed genes for bulk RNA sequencing transcriptomics experiment, 3-week post-SBI non-lactating vs 3-week post-SBI lactating. Genes listed are differentially expressed in 3-week post-SBI lactating animals.

| **Gene** | **log2FoldChange** | **padj** |
| --- | --- | --- |
| Csn1s1 | 8.595297109 | 0.04876 |
| Csn1s2a | 8.205605017 | 0.017609 |
| Wap | 7.387031712 | 4.18E-05 |
| Isca2-ps1 | 4.892656864 | 0.005537 |
| Fanca | 3.577729956 | 7.88E-13 |
| Hist1h2ao | 2.305621739 | 0.007127 |
| NA | 2.179524764 | 0.035649 |
| Hist1h2ao | 2.082647389 | 2.32E-05 |
| Col7a1 | 1.950444327 | 6.50E-05 |
| RGD1565143 | 1.943865188 | 0.032541 |
| Rhou | 1.662261766 | 4.12E-14 |
| Prodh | 1.608006328 | 0.00404 |
| Sqor | 1.423974306 | 6.71E-07 |
| Ddc | 1.409698972 | 0.002347 |
| Cntfr | 1.404907465 | 1.15E-05 |
| Nfil3 | 1.400811792 | 8.62E-10 |
| NA | 1.396043589 | 0.045465 |
| Pgpep1l | 1.386736275 | 0.017884 |
| Oas1k | 1.380809876 | 0.048658 |
| Fkbp5 | 1.350086424 | 5.21E-10 |
| Lrrc66 | 1.33203423 | 0.030521 |
| Sema7a | 1.319176038 | 1.28E-11 |
| Kcnj12 | 1.267148347 | 5.54E-09 |
| Nupr2 | 1.243082874 | 0.005661 |
| Tmem100 | 1.235637157 | 1.62E-06 |
| Glul | 1.230676849 | 2.28E-12 |
| Spock2 | 1.17374097 | 0.007528 |
| Dusp8 | 1.166459359 | 3.40E-06 |
| Foxp3 | 1.160347161 | 0.016997 |
| Ppp1r1a | 1.144981895 | 1.99E-09 |
| Ppp1r3c | 1.132064553 | 2.30E-10 |
| Fmo2 | 1.11344134 | 1.28E-06 |
| Arhgef26 | 1.110816961 | 0.02112 |
| Smyd2 | 1.098444249 | 1.86E-16 |
| Endou | 1.097942473 | 0.035623 |
| Klhl34 | 1.078981668 | 0.000689 |
| NA | 1.07356658 | 0.000223 |
| Clu | 1.05495521 | 1.80E-15 |
| Scx | 1.052527328 | 9.56E-07 |
| Cebpd | 1.026916404 | 0.00086 |
| Ccdc158 | 1.02026872 | 0.023633 |
| Il15 | 1.017741257 | 1.28E-06 |
| H4f3 | 1.009948033 | 0.006563 |
| Epb41l5 | 1.004621176 | 4.55E-06 |

**Experimental Section/Methods for Supplemental Figures:**

**Immunohistochemistry:**

CD68, CD163 and DAPI staining:

Tissue was fixed in acetone for 10 minutes. After PBS rinses, slides were incubated in blocking buffer (5% goat serum, 1% BSA, 0.05% Triton X-100 in PBS) for 2 hours and incubated with primary antibodies overnight at 4°C in blocking buffer (1:100 CD68, Abcam Cat#283654 and 1:250 CD163, Biorad Cat#MCA342R). After PBS rinses, slides were incubated with secondary antibodies for 1 hour at room temperature (1:500, Alexa Fluor 546 goat anti-rabbit IgG and Alexa Flour 647 goat anti mouse IgG).

Laminin, Myogenin, and DAPI staining:

Slides were fixed in 4% paraformaldehyde for 15 minutes. After PBS rinses, slides were incubated with blocking buffer (20% goat serum, 0.03% Triton X-100 in PBS) for 30 minutes followed by overnight primary incubation with laminin antibody (1:200, Sigma-Aldrich, St. Louis, MO). Slides were then incubated with secondary antibody (1:500, Alexa Fluor 546 goat anti-rabbit IgG) for 1 hour at room temperature. After PBS rinses, slides were fixed in 4% paraformaldehyde for 15 minutes, rinsed with PBS and then underwent antigen retrieval (Antigen unmasking solution, citric acid based) with an IHC-Tek Epitope Retrieval Steamer for 15 minutes. Slides were then incubated with blocking buffer for 1 hour at room temperature and then in primary antibody overnight for myogenin (1:200, BD Pharmingen, Cat#556358). Slides were rinsed with PBS and incubated with secondary antibody for 2 hours at room temperature (Alexa Flour 647 goat anti mouse IgG).

All slides were incubated with DAPI (1:10000) for 10 minutes to identify nuclei and mounted with Fluoromount and a coverslip. Slides were imaged with an Olympus VS200 Slide Scanner.

**Immunohistochemistry Analysis:**

For all immunohistochemistry analysis, investigators were blinded to the group identity.

*For CD68 and CD163 density quantification:*

For each animal analyzed, two stitched 20x images of the full PCa muscle tissue section near the origin were taken for quantification. QuPath software was used to select CD68+ and CD163+ cells co-localized with DAPI above a consistent threshold value. Area of the tissue section was taken for normalization.

*For MyoG quantification:*

For each animal analyzed, multiple stitched 20x image of the full PCa muscle tissue section near the origin was taken for quantification. QuPath software was used to select MyoG+ cells co-localized with DAPI above a consistent threshold value. Injured area of the tissue section was taken for normalization.
